# Rewiring of the Human Mitochondrial Interactome during Neuronal Reprogramming Reveals Regulators of the Respirasome and Neurogenesis

**DOI:** 10.1016/j.isci.2019.08.057

**Published:** 2019-09-04

**Authors:** Mohamed Taha Moutaoufik, Ramy Malty, Shahreen Amin, Qingzhou Zhang, Sadhna Phanse, Alla Gagarinova, Mara Zilocchi, Larissa Hoell, Zoran Minic, Maria Gagarinova, Hiroyuki Aoki, Jocelyn Stockwell, Matthew Jessulat, Florian Goebels, Kirsten Broderick, Nichollas E. Scott, James Vlasblom, Gabriel Musso, Bhanu Prasad, Eleonora Lamantea, Barbara Garavaglia, Alex Rajput, Kei Murayama, Yasushi Okazaki, Leonard J. Foster, Gary D. Bader, Francisco S. Cayabyab, Mohan Babu

**Affiliations:** 1 Department of Biochemistry, University of Regina, Regina, SK S4S 0A2, Canada; 2 Department of Biochemistry, University of Saskatchewan, Saskatoon, SK S7N 5E5, Canada; 3 Department of Surgery, Neuroscience Research Group, College of Medicine, University of Saskatchewan, Saskatoon, SK S7N 5E5, Canada; 4 The Donnelly Centre, University of Toronto, Toronto, ON M5S 3E1, Canada; 5 Department of Biochemistry and Molecular Biology, University of British Columbia, Vancouver, BC V6T 1Z3, Canada; 6 Department of Medicine, Harvard Medical School and Cardiovascular Division, Brigham and Women's Hospital, Boston, MA 02115, USA; 7 Department of Medicine, Regina Qu'Appelle Health Region, Regina, SK S4P 0W5, Canada; 8 Medical Genetics and Neurogenetics Unit, Fondazione IRCCS Instituto Neurologico Carlo Besta, via L. Temolo, 4, 20126 Milan, Italy; 9 Department of Medicine, Division of Neurology, College of Medicine, University of Saskatchewan, Saskatoon, SK S7N 5E5, Canada; 10 Department of Metabolism, Chiba Children's Hospital, 579-1 Heta-cho, Midori, Chiba 266-0007, Japan; 11 Graduate School of Medicine, Intractable Disease Research Center, Juntendo University, Hongo 2-1-1, Bunkyo-ku, Tokyo 113-8421, Japan

**Keywords:** Biological Sciences, Developmental Neuroscience, Developmental Biology, Proteomics

## Abstract

Mitochondrial protein (MP) assemblies undergo alterations during neurogenesis, a complex process vital in brain homeostasis and disease. Yet which MP assemblies remodel during differentiation remains unclear. Here, using mass spectrometry-based co-fractionation profiles and phosphoproteomics, we generated mitochondrial interaction maps of human pluripotent embryonal carcinoma stem cells and differentiated neuronal-like cells, which presented as two discrete cell populations by single-cell RNA sequencing. The resulting networks, encompassing 6,442 high-quality associations among 600 MPs, revealed widespread changes in mitochondrial interactions and site-specific phosphorylation during neuronal differentiation. By leveraging the networks, we show the orphan C20orf24 as a respirasome assembly factor whose disruption markedly reduces respiratory chain activity in patients deficient in complex IV. We also find that a heme-containing neurotrophic factor, neuron-derived neurotrophic factor [NENF], couples with Parkinson disease-related proteins to promote neurotrophic activity. Our results provide insights into the dynamic reorganization of mitochondrial networks during neuronal differentiation and highlights mechanisms for MPs in respirasome, neuronal function, and mitochondrial diseases.

## Highlights

• Rewiring of mitochondrial (mt) protein interaction network in distinct cell states
• Dramatic changes in site-specific phosphorylation during neuronal differentiation
• C20orf24 is a respirasome assembly factor depleted in patients deficient in CIV
• NENF binding with DJ-1/PINK1 promotes neurotrophic activity and neuronal survival

## Graphical Abstract

**Figure:**
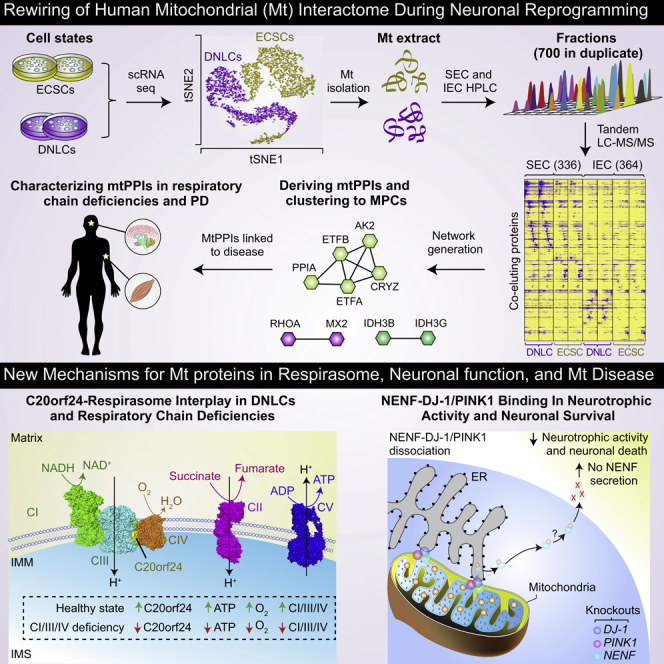

## Introduction

Mitochondria (mt) are dynamic organelles crucial for a number of essential cellular functions in neurons, including oxidative phosphorylation (OXPHOS), neuronal differentiation, and synapse formation (Nunnari and Suomalainen, 2012). Disruptions of mt functions can cause neuronal degeneration, leading to rare inherited metabolic (e.g., complex IV [CIV] or cytochrome *c* oxidase deficiency) or neurodegenerative (e.g., Parkinson disease [PD]) disorders (DiMauro and Schon, 2008). In normally functioning neurons, mt are crucial for neurogenesis, a dynamic process in which neural stem cells differentiate into neurons via a neurogenic gene expression program (Khacho et al., 2018). Conversely, the decline in neurogenesis leads to cognitive impairment associated with various degenerative disorders, and impaired mt may contribute to such deterioration (Fernandez et al., 2019, Khacho et al., 2018); however, the underlying mechanisms triggering these changes are poorly understood.

Global changes in gene expression (Busskamp et al., 2014) and proteome dynamics (Frese et al., 2017) have been observed across various stages of neuronal development in multiple cell types. Large-scale protein-protein interaction (PPI) networks generated by several proteomic methods (Hein et al., 2015, Huttlin et al., 2017, Wan et al., 2015) from whole cell, nuclear, or cytosolic extracts have provided a glimpse of the stably associated human complexome in non-neuronal cells. Yet, the physiological functions of mt proteins (MPs), as well as the organization of the full repertoire of cell-context-dependent, native human mtPPIs and resulting multiprotein complexes (MPCs) before and after neuronal differentiation are far from complete.

Accumulating evidence suggests that post-translational modifications (PTMs), including phosphorylation or dephosphorylation, regulate many aspects of mt processes (Grimsrud et al., 2012). Mass spectrometry (MS)-based proteomics has allowed the identification of the human mt proteome that is phosphorylated, as well as mt kinases that phosphorylate a number of different cellular protein substrates, and enabled the monitoring of changes in the phosphoproteome of human embryonic stem cells upon differentiation (Grimsrud et al., 2012, Van Hoof et al., 2009). Although these efforts in tissues and cell lines have enhanced our knowledge of the regulation of human proteins by PTMs, much remains to be learned about alterations in phosphorylation during neuronal differentiation. This includes how phosphorylation sites are distributed within MP complexes and which sites are targeted by mt kinases during neuronal differentiation.

Here, we address these gaps by performing an extensive biochemical fractionation (BF) with in-depth MS profiling in both mt extracts of cultured human NTera2 embryonal carcinoma stem cells (ECSCs or undifferentiated state) and retinoic acid (RA)-induced differentiated neuronal-like cells (DNLCs), two cell populations differentiable using single-cell RNA sequencing (scRNA-seq). The resulting network reveals that the majority of observed native mtPPIs were previously unreported and undergo considerable changes upon differentiation. Also, phosphoproteome characterization in the mt extracts from ECSCs and DNLCs shows a sizable fraction of MPs to be phosphorylated at serine residues and that the activity of mt pyruvate dehydrogenase E1α 2 subunit (PDHA2), phosphorylated on S291/S293 residues in ECSCs, is increased in DNLCs via dephosphorylation.

By leveraging the high-quality mtPPI network, we provide evidence that the orphan MP C20orf24, which has a less frequent heterozygous 3′ UTR variant in patients with mt respiratory chain deficiencies, functions as an assembly factor, causing a marked reduction in respirasome levels when disrupted. As well, we establish that the binding between a neuron-derived neurotrophic factor (NENF) and the PD-associated proteins (DJ-1/PARK7, PINK1), required for loading heme from mt, enhances neurotrophic activity to promote neuronal survival. Overall, this experimentally derived catalog of human mtPPIs assembled during the reprogramming of ECSCs to DNLCs will enhance our understanding of the functional significance of the mt in the intricate process of human neurogenesis and in the manifestation of mt diseases.

## Results

### BF/MS Co-elution Profiles from mt Extracts of NTera2 ECSCs and RA-Induced DNLCs

**Figure 1:**
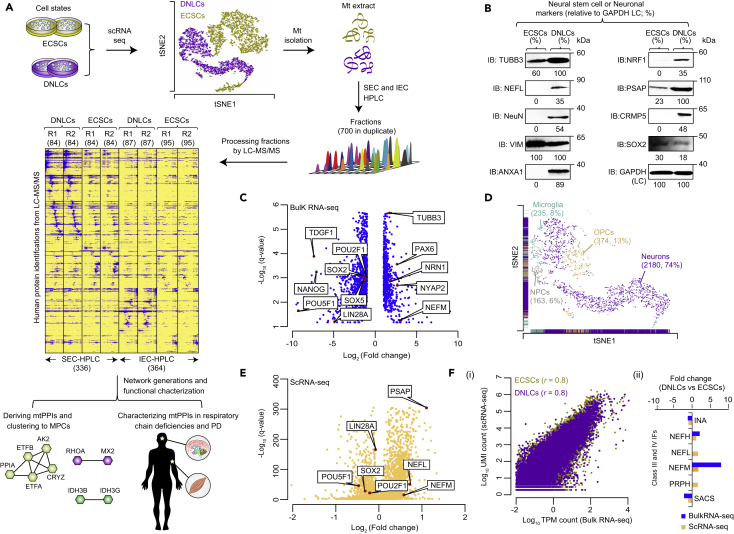
Experimental Workflow and Characterization of Cell States (A) BF/MS methodology to identify mtPPI changes (Tables S2 and S3) in ECSCs and DNLCs, which was characterized by scRNA-seq showing clear cell state separation by t-SNE (t-distributed stochastic neighbor embedding) plot. Heatmaps of co-eluting protein profiles of SEC- or IEC-high-performance liquid chromatography recorded by MS (unique peptide counts in blue) in duplicate experiments of each cell state. Numbers in parentheses indicate total fractions from each technique. (B) Immunoblotting (IB) showing the expression of indicated stemness/neuronal markers with respective protein-specific antibodies in ECSCs and DNLCs. (C and E) Volcano plots representing significant transcripts altered in DNLCs over ECSCs. Red dots indicate significantly up- or downregulated genes in DNLCs. Fold change was determined based on average TPM (transcripts per million; C) or UMI (unique molecular identifier; E) counts for each significant transcript in DNLCs over ECSCs. Q-value, false discovery rate (corrected by Benjamini-Hochberg)-adjusted p value. (D) t-SNE plot of 2,952 DNLCs, annotated by cell type identity. OPC, oligodendrocyte precursor cells; NPC, neural progenitor cells. (F) Scatterplot of log_10_ values (TPM or UMI counts) of all genes in each cell state (i), and transcriptional changes (fold change; DNLCs versus ECSCs) for classes III and IV intermediate filament (IF; ii) genes measured in RNA-seq and scRNA-seq. See also Figure S1 and Table S1.

To establish a map of native human mt macromolecular assemblies involved in neurogenesis, we applied our BF/MS strategy (Havugimana et al., 2012) to mt extracts isolated from chemically cross-linked (i.e., dithiobis-succinimidyl propionate, which allows identification of weak or transient PPIs; Malty et al., 2017) cultures of NTera2 ECSCs and DNLCs (Figure 1A). This human cell line was chosen because it is widely used in the study of neurogenesis and neurodegenerative disorders as an attractive progenitor that retains many human embryonic stem cell features, with the capacity to generate DNLCs (Gonzalez-Burguera et al., 2016). Besides confirming the expression of stemness and/or neuronal markers in ECSCs and DNLCs by immunoblotting (Figure 1B), immunofluorescence assays exhibited typical neuronal morphology for TAU-positive axons and MAP2-positive dendrites in DNLCs (Figures S1A and S1B).

We next assessed the global changes in transcriptome profiles during differentiation by performing bulk RNA sequencing (RNA-seq) in biological triplicates from ECSCs and DNLCs. The average correlation of transcriptomic profiles between replicates in each cell state was high (*r* = 0.99; Figure S1C), indicating good reproducibility. In total, we found that 10% (1,889) of the total transcripts (19,587) in DNLCs were significantly (*q* ≤ 0.01; Table S1) altered with a more than 2-fold change in expression when compared with ECSCs, resulting in 1,176 downregulated and 713 upregulated genes. Key pluripotency (*POU5F1*, *POU2F1*, *NANOG*, *TDGF1*, *LIN28A*) and stem cell maintenance (*SOX2*, *SOX5*) genes were downregulated, whereas neuronal cytoskeletal elements (*NEFM*, *TUBB3*) or regulators of neural precursors (*PAX6*, *NYAP2*, *NRN1*) exhibited an increase (3.4- to 8.1-fold, *q* = 4.5 × 10^−2^ to 1.7 × 10^−6^) in transcription (Figure 1C). These events suggest that NTera2 cells can faithfully model *in vivo* neuronal differentiation.

As incomplete differentiation from ECSCs to DNLCs or other causes of cell heterogeneity within a population of DNLCs can confound observation of interactions that are cell type specific, we performed scRNA-seq on live DNLCs relative to ECSCs and examined the gene expression dynamics of individual subpopulations. After processing the cells for quality control (see Transparent Methods), the sequenced data showed an average depth of 76,473 reads per cell and detected a median of 3,831 genes per cell. Cells analyzed in a two-dimensional t-distributed stochastic neighbor embedding (Figure 1A) plot showed a clear separation of cell states as two discrete populations, with 3,631 single cells from ECSCs and 2,952 from DNLCs.

Although RA exposure to ECSCs essentially facilitates irreversible differentiation, like any other differentiated cell lines, NTera2 DNLCs contain a mixed populations of neurons and other cell types. We therefore used previously described marker genes (Lake et al., 2016, Zhong et al., 2018) to predict cell type identity for all DNLCs, resulting in four transcriptionally distinct clusters (Table S1). Our analysis showed that three-fourths (2,180, 74%; Figure 1D) of the cells within the DNLCs correspond to the neuronal cell type (at 95% confidence by hypergeometric test); the rest identified as microglia (235, 8%), oligodendrocyte precursor (374, 13%), or neural progenitor (163, 6%) cells, suggesting that the interactions we identify in DNLCs will tend to be characteristic of the neuronal cell type.

We then performed differential gene expression analysis between DNLCs and ECSCs and identified 7,023 genes with significant (*q* ≤ 0.01; Table S1) differences in expression in DNLCs (4,503 upregulated and 2,520 downregulated) when compared with ECSCs (Figures 1E and S1D). As for marker genes from immunoblotting or RNA-seq for ECSCs and DNLCs (Figures 1B and 1C), the overall average expression level of single cells correlated well (*r* = 0.8) with the bulk cell populations (Figure 1F). An illustrative example is the involvement of an ARSACS (autosomal recessive spastic ataxia of Charlevoix-Saguenay)-causing *SACS* gene in the regulation of class III (*PRPH*) and IV (*NF-L/M/H*; *INA*) intermediate filament genes expressed in neurons (Gentil et al., 2019). Consistent with this observation, bulk RNA-seq and scRNA-seq showed an increased expression of classes III and IV genes in DNLCs, except *SACS* and *INA*, which exhibited decreased expression (Figure 1F), revealing a cell type-specific outcome.

After confirming morphological and transcriptional changes as expected in ECSCs and DNLCs, we sought to co-fractionate the stably interacting proteins by performing BFs using complementary size-exclusion and ion-exchange chromatographic (SEC, IEC) separation techniques (Figure 1A). A total of 700 distinct fractions were collected in duplicate from the cross-linked mt extracts of four fractionation experiments (two ECSCs, two DNLCs). These fractions were then subjected to MS to define MP complex membership. Examination of the co-elution profiles and average correlation of proteins (peptide counts) detected by MS between replicate experiments suggested high reproducibility (*r* = 0.99; Figures 1A and S2A). As expected, elution profiles were also consistent with average molecular weights and isoelectric points by SEC and IEC methods (Figure S2B), respectively, reinforcing the utility of BF/MS approach.

### Scoring and Validating Human mtPPIs in ECSCs and DNLCs

To generate high-quality mtPPIs in each cell state, the tandem MS spectra from each replicate chromatographic fraction was searched against reference human protein sequences using Sequest and several alternate search engines (X! Tandem, MS-GF+, Comet) to increase the sensitivity and accuracy of peptide identification. The resulting peptide-spectral matches were integrated into a single probability score using MSblender (Kwon et al., 2011) and then filtered to a 0.1% protein-level false discovery rate, with two or more distinct peptides used to define human proteins that reliably co-elute in each fraction. The chromatographic profiles of proteins co-eluted across collected fractions from both replicates were measured using three complementary scoring procedures (Pearson correlation coefficient, weighted cross-correlation, co-apex score, Havugimana et al., 2012) to identify pairwise protein associations. To maximize coverage and accuracy (Figure S2C), we integrated the correlation and co-apex scores from different separation techniques into a single unified log likelihood score (LLS) for each putatively interacting protein pair in ECSCs (ΣLLS_ECSCs_) and DNLCs (ΣLLS_DNLCs_), respectively.

**Figure 2:**
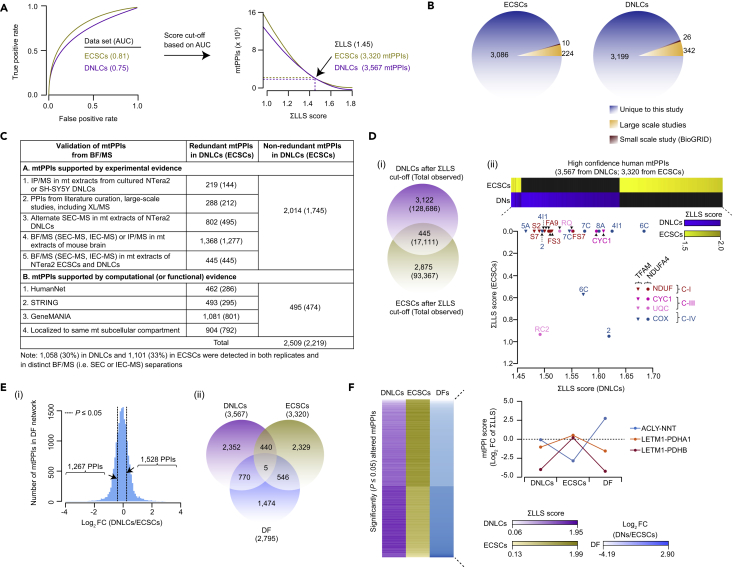
Benchmarking and Remodeling of mtPPIs in ECSCs and DNLCs (A) Performance measures using AUC (area under the receiver operating characteristic curve) analysis based on estimates of true- and false-positive rates for scored mtPPIs against reference CORUM protein complexes containing mtPPIs (top). Cumulative ΣLLS score (bottom; threshold based on AUC) was computed by combining LLS score derived from SEC and IEC fractions. (B) Overlap of high-confidence mtPPIs from ECSC and DNLC networks against the large- or small-scale (curated in BioGRID database) studies. (C) Experimental and computational evidence supporting mtPPIs from each cell state. (D) High-confidence (and total) mtPPIs common or specific to ECSC or DNLC networks (i). Association of CI subunit (NDUFA4) or TFAM with the components of the respirasome (ii) along with their ΣLLS scores in two static networks. (E) *Z* score distribution (i) of log_2_ fold change (FC) for differential (DF) PPIs filtered at p value ≤ 0.05 (|*Z score*| ≥ 1.96) with tails indicating significant interactions. Venn diagram (ii) showing the overlap of significant mtPPIs in static and DF networks. (F) Heatmap displaying significantly altered mtPPIs during differentiation (left) is shown with illustrative examples (right) for static and DF profiles. See also Figures S2 and S3, and Table S2.

High-confidence mtPPIs for each cell state were derived by eliminating associations below a stringent cutoff (ΣLLS ≤1.45), where most of the reference mtPPIs curated in CORUM human protein complexes (Ruepp et al., 2010) were recovered, as evaluated using area under the receiver operating characteristic curve (Figure 2A). After employing an appropriate threshold, we considered as physiologically relevant the interactions between MPs, and between the cytosolic and outer mt membrane (OMM) proteins (Walther and Rapaport, 2009), for defining two static mtPPI networks. These included 3,320 interactions (2,973 between MPs; 347 between cytosolic and OMM) among 408 unique human MPs in ECSCs and 3,567 (3,233 between MPs; 334 between cytosolic and OMM) interactions among 467 MPs in DNLCs, covering 36% (600 of 1,672 MPs) of the estimated human mt proteome (Table S2).

In addition to confirming the physical associations observed in previous large-scale interaction studies in humans and other metazoans (Floyd et al., 2016, Havugimana et al., 2012, Hein et al., 2015, Huttlin et al., 2017, Liu et al., 2018, Malty et al., 2017, Schweppe et al., 2017, Wan et al., 2015) or small-scale biochemical experiments (BioGRID), we found that most of the mtPPIs in ECSCs (3,086, 93%) and DNLCs (3,199, 90%) had not been previously reported (Figure 2B). Notably, these PPIs encompassed an order of magnitude more MPs (Figure S2D) than our recent study (Malty et al., 2017) performed in differentiated SH-SY5Y human neuronal cells. The overall reliability, data quality, and biological relevance of mtPPIs in the filtered static (ECSCs, DNLCs) networks was further assessed by composites of various experimental and computational sources (Figure 2C; Table S2). Half (1,745, 53% in ECSCs; 2,014, 56% in DNLCs) of our observed interactions were verified in other cell types and/or mouse brain, or by independent experimental evaluation. These include mtPPIs supported by (1) immunoprecipitation (IP) combined with MS (IP/MS) using protein-specific antibodies in the NTera2 or SH-SY5Y DNLCs or mouse brain, (2) BF/MS in mouse brain or in both NTera2 cell states, (3) alternate SEC separation and MS in NTera2 DNLCs, and (4) previous high-throughput or literature-curated interaction reports (withholding PPIs from CORUM reference set).

The validity of the remaining mtPPIs from BF/MS was further independently verified by computational approaches. Specifically, less than one-quarter (474, 14% in ECSCs; 495, 14% in DNLCs) of our interactors have shared functional associations predicted in HumanNet (Lee et al., 2011), STRING (Szklarczyk et al., 2017), or GeneMANIA (Warde-Farley et al., 2010), or are known or predicted to localize to the same mt subcellular compartment. Altogether, two-thirds (2,219, 67% in ECSCs; 2,509, 70% in DNLCs) of the total, non-redundant physical associations confirmed by these experimental or computational sources are consistent with the validation rates seen for human soluble PPIs (Havugimana et al., 2012).

When benchmarked against literature-curated interactors (CORUM), the performance measures of our mtPPI networks (using 5-fold cross-validation) showed high sensitivity and specificity (Figure S2E), when compared with previous human interaction datasets (Floyd et al., 2016, Havugimana et al., 2012, Hein et al., 2015, Huttlin et al., 2017, Wan et al., 2015). The putative interacting MPs were also significantly (4.7 × 10^−64^ < p < 1.9 × 10^−59^) enriched for shared phenotypic annotations and higher (7.0 × 10^−52^ < p < 1.0 × 10^−14^) functional coherence and similarity (based on Gene Ontology annotations) compared with existing large-scale PPI studies (Figure S3A). The ranked list of mtPPIs within the top or bottom 20th percentile of ΣLLS scores showed significant (5.3 × 10^−46^ < p < 2.6 × 10^−33^) enrichment for membership in the same CORUM protein complex (Figure S3B), indicating that the subunits of these complexes were more likely to co-elute in the same biochemical fractions. MP interactors also tended to show enrichment (3.0 × 10^−6^ < p < 5.0 × 10^−2^) for shared Pfam domains (Figure S3C; Table S2). This includes the cytochrome *b*_5_ domain-containing proteins that function as electron carriers for membrane-bound oxygenases, and the heat shock protein (HSP70, HSP90) domain families involved in proteostasis and quality control. Finally, stably interacting proteins that co-fractionated together were strongly (6.7 × 10^−131^ < p < 2.2 × 10^−14^; Figure S3D) co-expressed or co-translated in NTera2 (ECSCs, DNLCs) or mouse brain (Sharma et al., 2015, Zhang et al., 2014) and exhibited significantly (8.4 × 10^−71^ < p < 5.6 × 10^−42^) positively correlated mRNA co-expression profiles (Figure S3E) in human cortical neurons (van de Leemput et al., 2014). These findings suggest a propensity of mtPPIs to have coordinated expression in neurons or brain regions.

### mtPPI Network Reflects Dynamic Changes in NTera2 ECSCs and DNLCs

Although the putative physical interactions observed in both networks appeared to be corroborated by transcriptional evidence (Figure S3F), the two interaction networks were markedly different at our chosen ΣLLS threshold. Specifically, there were more mtPPIs unique to each cell state (3,122 in DNLCs; 2,875 in ECSCs) than shared in common (Figure 2D). For example, binding of the mt-encoded NADH dehydrogenase transmembrane subunit of the respiratory complex I [CI] (NDUFA4) with its members (NDUFA9, FS3, FS7), as well as with the constituents of complex III [CIII] (CYC1, UQCRC2, UQCRQ) and complex IV [CIV] (COX2, 4I1, 6C, 7C, 8A) in DNLCs supports the notion that NDUFA4 may coordinate its function as an assembly factor for respirasomes (Kadenbach, 2017) during neuronal differentiation. Similarly, the association of mt transcription factor A (TFAM) with the subunits of respiratory complexes lends support to the suggestion that TFAM regulates the respiratory chain function as it is required for the expression of the components of mt complexes (Larsson et al., 1998, Masand et al., 2018). The transitioning of mtPPIs during differentiation was further supported by the high degree of connectivity (≥50 PPIs) of MPs that participate in mt pathways, including respiration and metabolism (Figure S4A). As these findings imply highly differential underlying networks, we next examined the validity of these observed differences.

To characterize the changes in mtPPI networks between cell states, we constructed a differential (DF) network by computing a fold change for each protein pair using the ΣLLS score from DNLCs relative to ECSCs (ΣLLS_DNLCs_/ΣLLS_ECSCs_). Given that DF interactome mapping can identify interactions that undergo alterations reliant on cell or tissue type, conditions, and disease state or other variants (Hosp et al., 2015, Ideker and Krogan, 2012), we focused on stringently identified differences. Based on the null distribution of the scored data, we applied thresholds corresponding to two standard deviations (p ≤ 0.05), resulting in 2,795 mtPPIs that were significantly DF in ECSC- or DNLC-specific function (Figure 2E; Table S2). Half (51%, 1,528) of the DF mtPPIs with low ΣLLS scores in the static ECSC network became pronounced in DNLCs upon RA induction (Table S2). After filtering the significant DF network with a fold change cutoff of 2.0, 225 MP pairs were identified as likely to interact in one network but unlikely in the other (Figure 2F). For example, a high-confidence (6.9-fold) physical association between nicotinamide nucleotide transhydrogenase and ATP-citrate lyase in the DF network was reconcilable with the prediction from STRING, which suggested a functional link based on the role of these proteins in glutamine metabolism (Alberghina and Gaglio, 2014). In contrast, interaction (1.6- to 4.2-fold) between the inner mt membrane (IMM) protein (LETM1) and the mt matrix pyruvate dehydrogenase (PDH) complex subunits (PDHA1, PDHB) in ECSCs indicates that a physical coupling of these proteins may be relevant to glucose metabolism (Durigon et al., 2018, Jiang et al., 2013). These patterns in the alterations of mtPPIs in distinct cell states further suggest that many testable mechanistic hypotheses can be mined from the static or DF networks.

As proteins that associate physically tend to have similar biological functions (Oliver, 2000), the mtPPI networks from both cell states were examined to gain insight into the roles of orphan MPs (i.e., those with unclear biological significance) based on their association with annotated components. We found seven orphans in the high-confidence static networks to be connected through 132 associations. The number of PPIs identified for each orphan in the mt networks also varied considerably, ranging from 1 (e.g., C1orf64) to 53 (e.g., C20orf24), with many MPs in the DN network interacting with an orphan protein. Among the 132 PPIs with orphan proteins, nearly three-fourths (94, 71%) were altered in response to RA-induced neuronal differentiation (Figure S4B). Overall, these observations show wide reprogramming of mtPPIs during neuronal differentiation, vastly expanding the existing mt interactome.

### Endogenous MPCs Are Altered during Neuronal Differentiation

Given that the topological analysis of the PPI network can reveal clusters of densely connected proteins, we examined how macromolecular protein assemblies are reconfigured in response to RA-induced differentiation. As seen for scale-free PPI maps (Havugimana et al., 2012, Malty et al., 2017), interacting proteins in the DNLC network followed a power-law distribution, with most proteins associated to each other within five or fewer degrees of separation (Figure S4C).

**Figure 3:**
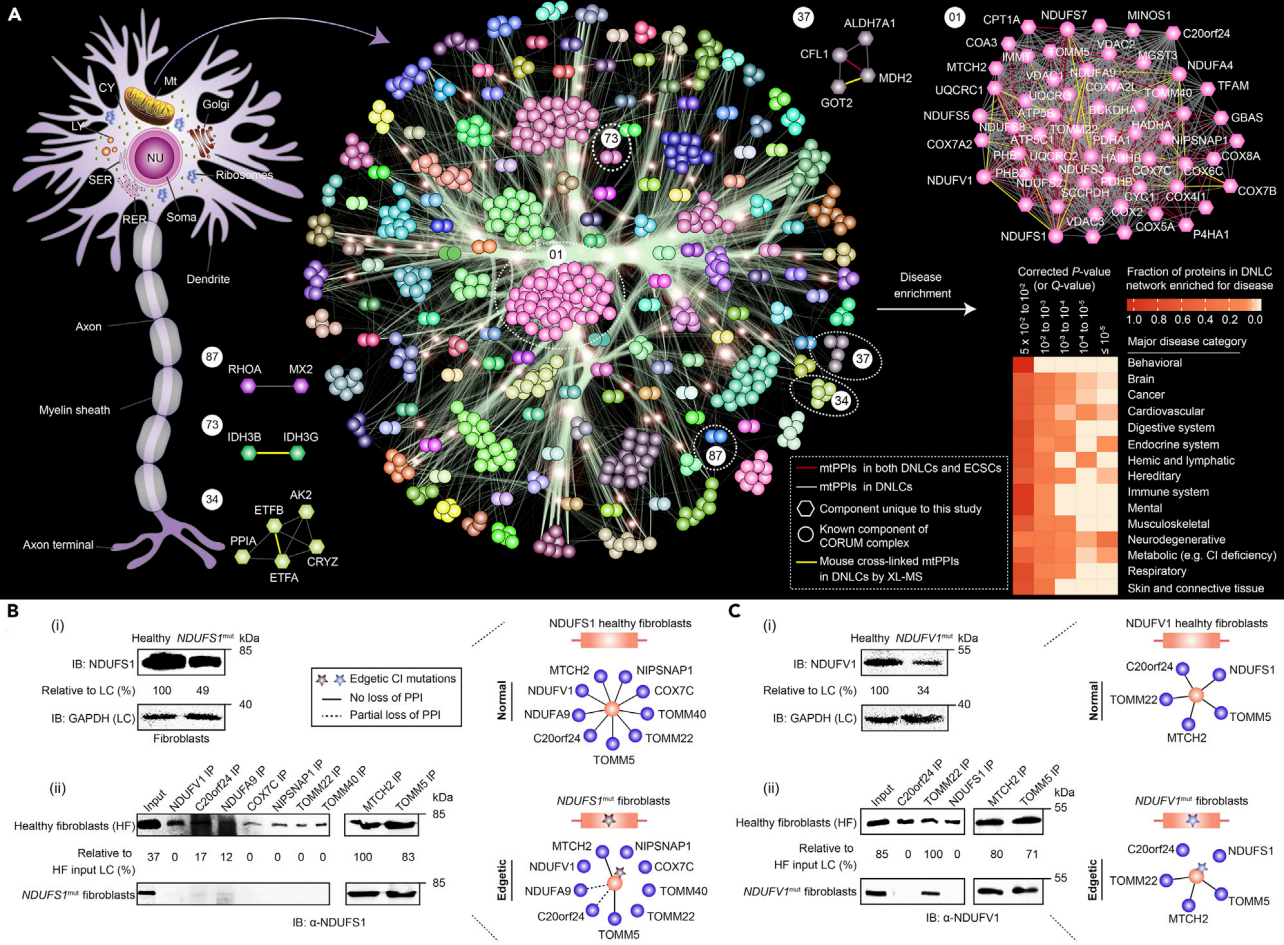
Human mt MPCs in DNLCs and Their Interacting Proteins to CI Deficiency (A) Schematic of the putative MP assemblies and representative MPCs are indicated using different colored circles, and mtPPIs (edges or lines) present in both cell states (red) and specific to DNLCs (gray) are shown along with those identified by cross-linking (XL)-MS (yellow) in different mouse tissues; hexagonal nodes are unique to this study, and circles are known component of a CORUM complex. CY, cytoplasm; SER and RER, smooth and rough ER, respectively; LY, lysosome. Enrichment of mtPPIs from DNLC network linked to human diseases; false discovery rate (Benjamini-Hochberg correction)-adjusted p value (or *Q*-value) of the hypergeometric test. (B and C) NDUFS1 (B-i) or V1 (C-i) levels and perturbation of select PPI pairs in the fibroblasts of healthy individual and CI-deficient subject with *NDUF* mutations (B-ii, C-ii) immunoblotted (IB) with anti-NDUFS1 or VI antibody. Band intensities were normalized to GAPDH or healthy fibroblast (HF) input loading control (LC). Molecular masses (kDa) of marker proteins are indicated. See also Figure S4 and Table S3.

To systematically define complex membership in the static DNLC network, we employed the coreMethod (Leung et al., 2009), Markov clustering (Enright et al., 2002), and ClusterONE (Nepusz et al., 2012) algorithms. Comparison of the resulting protein clusters from each of the clustering methods to the literature-curated training set of protein complexes in CORUM showed that clusters generated using the coreMethod overlapped more substantially (p = 1.3 × 10^−13^) with known complexes (Figure S4D), and as a result, we focused on the coreMethod clusters from this point forward. The coreMethod produced 139 putative multiprotein groupings with at least one MP, including 72 heterodimers (Figure 3; Table S3). Proteins interacting in one-fourth (32, 23%) of these complexes derived from DNLCs also existed in ECSCs (Table S3), whereas one-third (40, 30%) matched the reported CORUM assemblies. The remaining 99 putative complexes have not been previously characterized (Figure S4E), signifying a valuable resource for exploring the mechanistic basis of the interactions in biological contexts.

We then interrogated the 139 MPCs by comparing them with two recent mt interactome maps generated in mouse tissues by cross-linking MS (Liu et al., 2018, Schweppe et al., 2017). Through ortholog mapping we identified 55 unique cross-linked interacting protein pairs in mouse that map to 13 of our identified MPCs (Table S3). For example, 27 mouse interactors were observed in the human mt respirasome complexes (CI/III/IV); however, the association of mt fatty acid β-oxidation of 3-hydroxyacyl-CoA dehydrogenase (HADHA, B) with these complexes, which is consistent with the related role of fatty acid β-oxidation protein products in OXPHOS complex biogenesis (Lim et al., 2018), has not been previously observed in DNLCs. Similarly, the cross-linking performed in mouse tissues connected mt glutamate oxaloacetate transaminase (GOT2) and malate dehydrogenase in DNLCs (MDH2), supporting their role in malate-aspartate shuttling for energy production (Yang et al., 2015). Although the mechanism behind the interactions remains to be determined, these results suggest that the cross-linked associations from mouse were enriched in orthologs to human proteins in close spatial proximity, and are generally consistent with the limited MPC relationships observed in the DNLC network.

Because MPC subunits with correlated interaction profiles can not only lend additional evidence for predicted MPCs but also predict functional relationships between proteins (Han et al., 2017), we calculated the correlation of interaction partners between all possible pairs of proteins present in the static ECSC and DNLC networks. Consistent with the shared functionality implied by their co-grouping, the interaction profiles for proteins within the same complexes in ECSCs or DNLCs were found to be significantly (p < 1.3 × 10^−86^ to p < 1.9 × 10^−7^) more positively correlated on average than protein pairs associated between complexes (Figures S5A and S5B). Interaction profile correlations also revealed many significantly (p ≤ 0.05) positively correlated protein pairs of the same complex to be involved in both cell states (Figures 3 and S5A). This includes the strong correlation (*r* = 0.4–0.9; Table S3) between the mt electron-transferring flavoprotein partners (ETFA, ETFB) that shuttle electrons to coenzyme Q (complex 34), as well as between the components of mt voltage-dependent anion channel (VDAC1, 2, 3) and protein translocases (TOMM5, TOMM40; complex 1), consistent with the requirement of TOMM in VDAC import (Kozjak-Pavlovic et al., 2007). Conversely, we also found positively correlated interaction profiles for MPC subunits that are preferentially detected in DNLCs (Table S3). An illustrative example is the highly correlated (*r* = 0.9) interaction profiles between the isocitrate dehydrogenase complex subunits (IDH3B, IDH3G; complex 73), as well as the positively correlated (*r* = 0.9) interacting protein profiles observed between the poorly characterized small RhoGTPase (RhoA) and MX dynamin-like GTPase (MX2; complex 87). These observations suggest that the protein pairs may have functions in neuronal processes.

### MPCs in DNLCs Reveal Interactions Linked to CI Deficiency and Other mt Disorders

Disease-associated alleles have been observed in the binding interface of interacting proteins, with disruption of protein associations cited as leading to disease progression (Sahni et al., 2015). We therefore examined the DNLC network to identify the interacting subunits of MPCs relevant to human diseases. Over half (267, 57%) of MPs that make up the interactions in the MPCs have links to a spectrum of mt disorders with ∼300 known disease-causing genes (Kremer et al., 2017) and are significantly enriched (p < 1 × 10^−5^ to p < 5 × 10^−2^) for multiple human disease annotations from DisGeNET (Pinero et al., 2017). One notable enrichment was the interacting proteins with associations to CI deficiency (Figure 3A), a rare inborn error of metabolism due to mutations in nuclear (or mt) genes encoding subunits of the human mt CI.

As an MPC consisting of CI subunits, *NDUFS1* and *V1,* was highly connected to 46 putatively interacting proteins in the DNLC network, we examined the potential relevance of these interactions to CI deficiency in isolated skin fibroblasts from CI-deficient children carrying pathogenic mutations (Transparent Methods) in *NDUFS1* and *V1* alleles. Although *NDUF* mutants were expressed at modest levels in the fibroblasts of patients with CI, their mutations perturbed two-thirds (9, 64%) of all (14) high-scoring tested interactions in the CI patient fibroblasts (Figures 3B and 3C). For example, mutation in the *NDUFS1* gene disrupted its interactions with OMM import receptors (*TOMM22*, *40*), consistent with the functional dependency between mt import and respiratory chain machineries (Kulawiak et al., 2013). As well, *NDUFS1* or *V1* mutations abrogated the ability of these genes to bind the subunits of CI and CIV, supporting the idea that mutations in the CI gene products may affect respirasome assembly (Floyd et al., 2016). Overall, our results suggest that known CI mutations preferentially induce edgetic perturbations.

### Phosphorylation within the MPC Reveals Cell State-Specific Differences

**Figure 4:**
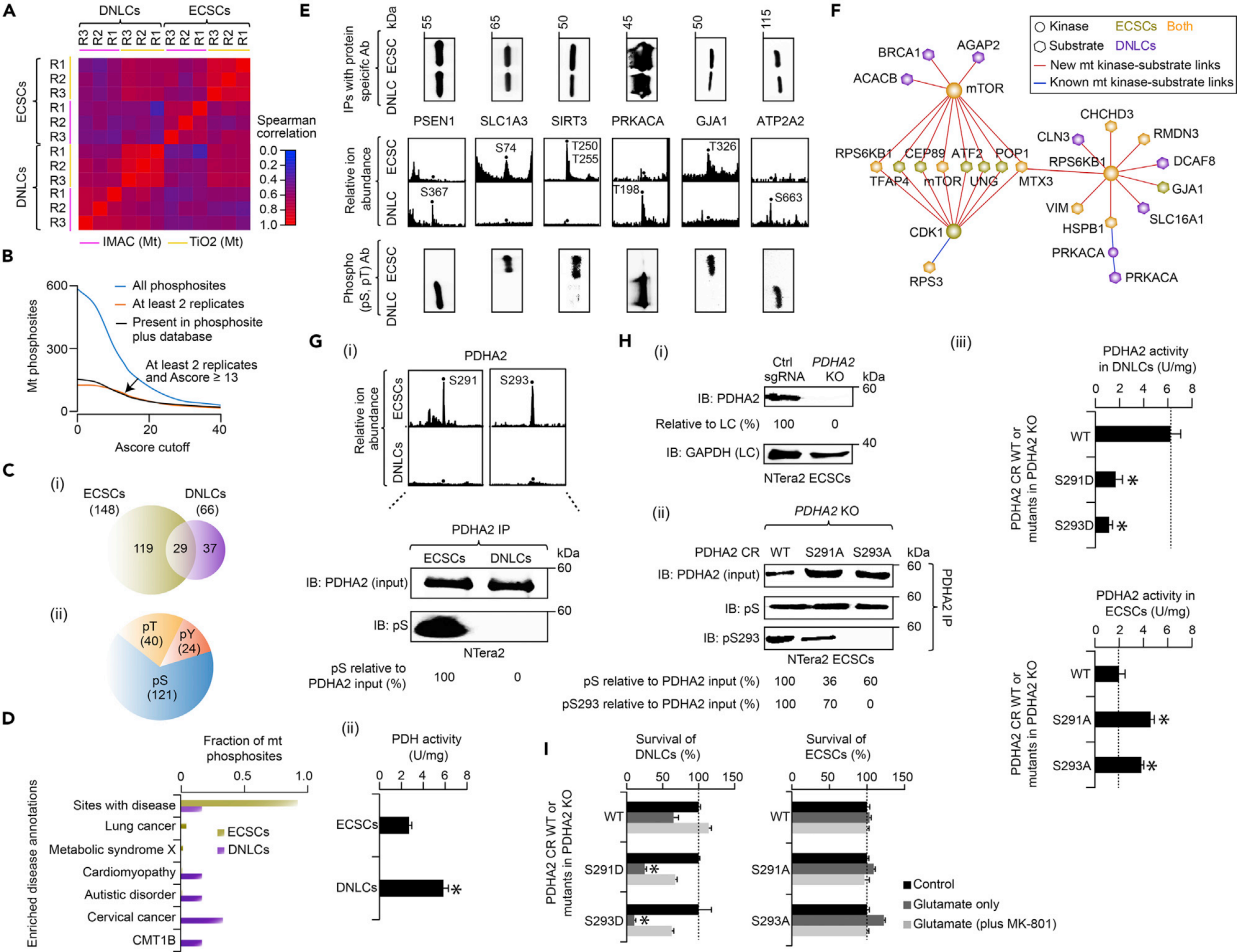
Identification of Phosphorylation Sites in ECSCs and DNLCs (A) Spearman correlation profiles clustered between phosphoproteome replicate experiments (using peptide counts for each phosphorylation site) from the mt extracts of ECSCs and DNLCs using the indicated phosphopeptide enrichment methods. (B) mt phosphosites in each cell state with various Ascore cutoff and replicate measurements. (C) Phosphosites specific or common to each cell state (i) and their localization (ii) on serine (pS), threonine (pT), and tyrosine (pY) residues. (D) Enrichment of all phosphosites and those associated with human diseases (few representative ones highlighted); false discovery rate (Benjamini-Hochberg correction)-adjusted p value (or *Q*-value) of the Fisher's exact test. (E) Relative ion abundance for proteins indicated with cell state-specific phosphosites were immunoprecipitated and probed with protein-specific and phospho (pS, pT) antibodies. (F) Kinase-substrate phosphorylation network (Table S4) in ECSCs and/or DNLCs is indicated with mt kinases (circle) and their mt substrates (hexagonal). (G) Relative ion abundance of PDHA2 phosphosites in ECSCs, and immunoprecipitates of PDHA2 immunoblotted (IB) with pS antibody (i). Band intensities normalized to PDHA2. PDH activity (ii) measured after 60 min of incubation with mt extracts from ECSCs or DNLCs. (H) PDHA2 (i) and pS or pS293 (ii) levels measured in control (ctrl) single-guide RNA (sgRNA) and *PDHA2* KO ECSCs, or in the immunoprecipitates of *PDHA2* KO ECSCs complemented with wild-type (WT) CRISPR-resistant (CR) copy of *PDHA2* and non-phosphorylatable mutants using anti-PDHA2 antibody were IB with indicated antibodies. Band intensities normalized to PDHA2. Activity of PDHA2 (iii) is shown after 60 min of incubation with the mt extracts of WT or *PDHA2* phosphomimetic and non-phosphorylatable mutants from DNLCs and ECSCs, respectively. (I) Survival of *PDHA2* mutants and WT ECSCs or DNLCs in control salt solution, glutamate (1 mM), or glutamate plus MK-801 (300 nM). Data for (G, H, and I) are mean ± SD (*n* = 3 biological replicates; *p ≤ 0.05 by Student's t test). See also Table S4.

PTMs such as phosphorylation of serine (pS), threonine (pT), and tyrosine (pY) modulate numerous functions, including for MPs involved in MPC formation, facilitation or disruption of PPIs, induction or elimination of enzyme activities, and alteration of protein conformations (Huttlin et al., 2010). We therefore hypothesized that there should be dramatic changes in site-specific phosphorylation before and after differentiation. To test this hypothesis, we surveyed the phosphorylation dynamics in mt extracts isolated from the cultured ECSCs and DNLCs, respectively. Phosphopeptides were enriched based on immobilized metal-affinity chromatography and titanium dioxide-metal oxide affinity chromatography, run in triplicate, and were then analyzed using high-resolution MS. The average correlation (*r* = 0.8) of phosphoproteome replicate experiments was high, and replicates were appropriately clustered by their cell state and phosphopeptide enrichment methods (Figure 4A).

After applying the Ascore algorithm with a confidence cutoff of 95% (Ascore ≥ 13), and retaining phosphorylated residues detected in two of the three replicates or in PhosphoSitePlus (a public protein phosphorylation repository; Figure 4B), we observed a total of 185 non-redundant phosphosites in 81 mt phosphoproteins (from a total of 190 mt phosphoproteins detected before filtering) that were localized to 121 serines, 40 threonines, and 24 tyrosines (Figure 4C). About one-quarter (38, 21%) of the phosphosites from ECSCs or DNLCs were enriched (*q* ≤ 0.05) for disease annotations including cancer, metabolic syndrome, neurological or neurodevelopmental disorders, and cardiomyopathy (Figure 4D; Table S4). Among the detected phosphosites, 30 were not previously described, and of the rest (155, 84%) that were found in the PhosphoSitePlus database, a majority (148, 95%; Table S4) have not been reported in the cell states we examined.

Most (156, 84%) of the phosphosites examined were also found to be altered between ECSCs and DNLCs (Figure 4C; Table S4). These include DN-specific phosphorylation on proteins involved in synaptic function (PSEN1 at S367), signaling (PRKACA at T198), and OXPHOS (ATP2A2 at S663, Y867). Conversely, we found several proteins to be phosphorylated only in ECSCs. These included a regulator of mt metabolism (T250/255, S253 of SIRT3), a gap junction protein functioning in stem cell maintenance (GJA1 at T326, S279/282/306/314/325/365, Y301/313), and glutamate transporter of proliferating stem cells (S74, Y71/75 of SLC1A3). A selection of cell state-specific phosphosites were confirmed by immunoblotting using antibodies that recognize phosphorylated motifs (Figure 4E). The phosphoproteomic data were then mapped onto the 139 MPCs to reveal how the interacting subunits are regulated by phosphorylation. We found 17 mt subunits in one-fifth (26, 19%; Table S4) of the MPCs to be phosphorylated during differentiation. These include MPCs containing the phosphosites on the interacting proteins of mt structure and function (CHCHD3), energy metabolism (BCKDHA, PDHA1, HACD3), and transport (VDAC1), reflecting modest capacity for post-transcriptional modification.

The dynamic phosphorylation events observed among the proteins in ECSCs or DNLCs led us to investigate which mt kinases carry out these modifications. We therefore specifically searched the mt kinases and their substrates localized in mt for all identified phosphosites by mapping known kinases defined in PhosphoSitePlus and high-confidence kinase-specific phosphosites predicted from the GPS 3.0 tool. By constructing a human mt kinase-substrate phosphorylation subnetwork in ECSCs and/or DNLCs, we could identify 3 known and 18 putative unexplored substrates for 4 different mt kinases (mTOR, CDK1, PRKACA, RPS6KB1), encompassing 31 kinase-substrate relationships (Figure 4F; Table S4). For example, a series of cell state-specific phosphorylation sites of potential substrates involved in mt and neuronal functions (ATF2, CEP89, MTX3, POP1, TFAP4, UNG, RPS6KB1) were predicted for two (mTOR, CDK1) known kinases, highlighting the intricate mt kinase-substrate relationships involved in the process of neuronal differentiation.

Among the mt phosphoproteins detected, we found two (S291, S293) phosphorylated serine residues on PDHA2 in ECSCs, but not in DNLCs. Although PDHA2 phosphosites have been reported in PhosphoSitePlus, little is known about their effects on PDHA2 activity during differentiation. Immunoblotting PDHA2 IPs with an anti-phosphoserine antibody in ECSCs and DNLCs confirmed a phosphorylated serine in ECSCs, and not in DNLCs. As well, we found that the mt PDH activity in DNLCs was increased when compared with ECSCs (Figure 4G). These observations led us to hypothesize that PDHA2, responsible for the conversion of pyruvate into acetyl coenzyme A, becomes dephosphorylated to increase its enzyme activity, presumably to cope with the increased energy requirements of NTera2 cells during neuronal differentiation.

To ascertain if the increased phosphorylation of serine residues in ECSCs was specific, we generated CRISPR-Cas9-mediated *PDHA2* knockouts (KOs) in ECSCs and complemented the cells with the wild-type CRISPR-resistant copy of *PDHA2* and phosphomimetic (*PDHA2* S291D, S293D) or non-phosphorylatable (*PDHA2* S291A, S293A) mutants to mimic constitutive phosphorylation and dephosphorylation, respectively. In contrast to wild-type, non-phosphorylatable mutants in ECSCs exhibited reduced phosphoserine levels using available antibodies for anti-phosphoserine or specific to PDHA2 residue phosphorylated at S293, indicating that the mutants were expressed and had the expected phosphorylation status (Figure 4H). Most notably, the non-phosphorylatable mutants in ECSCs showed a 2.0- to 2.3-fold increase in PDHA2 activity over wild-type, whereas in DNLCs the phosphomimetics significantly lowered (4- to 6-fold) the PDHA2 activity (Figure 4H). This modulation in the activity of PDHA2 by phosphorylation suggests a mechanism by which cells adapt to meet increased energy demands of neurons during differentiation. To examine this notion further, we hypothesized that the phosphomimetic *PDHA2* mutants would result in reduced survival of DNLCs in response to glutamate excitotoxicity (i.e., neuronal death caused by glutamate exposure) due to impairment in energy production. Intriguingly, exposure of phosphomimetic mutants to glutamate significantly reduced survival in DNLCs when compared with wild-type cells. This effect was specific as MK-801 (a glutamate receptor antagonist) rescued the DNLCs from glutamate cytotoxicity. Conversely, non-phosphorylatable mutants did not show any considerable effect on the ECSCs when exposed to glutamate with and without MK-801 (Figure 4I). Taken together, our results suggest that the PDHA2 dephosphorylation in DNLCs plays a role in enhancing energy production, which is required to protect from glutamate excitotoxicity, whereas phosphorylated PDHA2 is detrimental to DNLCs.

### Orphan Membership within MPCs in DNLCs Reveals C20orf24-Respirasome Interplay

**Figure 5:**
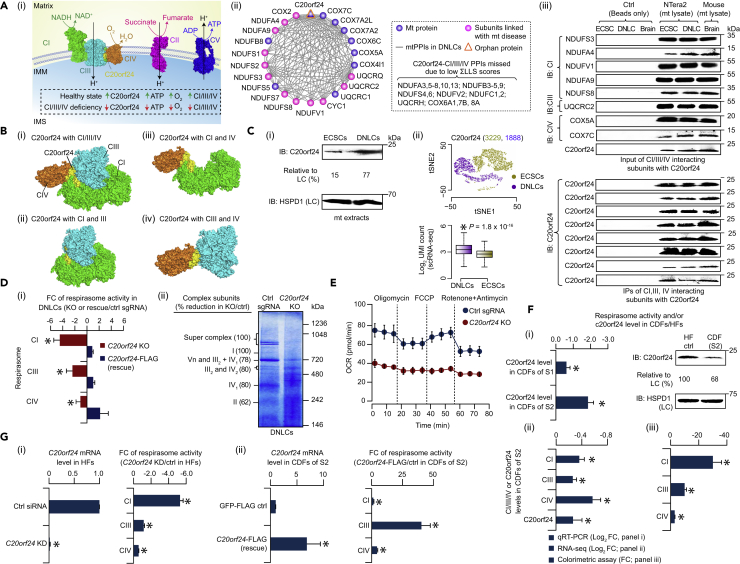
C20orf24 is Required for Respirasome Assembly (A) Model of C20orf24-respirasome interplay in healthy individuals versus patients with respiratory chain defects (i). Sub-network of C20orf24 link with CI/III/IV subunits in DNLCs (gray lines; ii). CI/III/IV-specific antibody immunoprecipitates and input from the mt extracts of ECSCs, DNLCs, and mouse brain immunoblotted (IB; iii) with antibody raised against C20orf24 and its interacting proteins; protein G beads alone (without antibody) served as negative control. (B) Docking of C20orf24 (yellow) on the human respirasome CI (green), CIII (cyan), and CIV (orange) structures (i) is shown as a zoom-in where C20orf24 can be seen at the interface with indicated structures of respiratory complex subunits (ii–iv). (C) Endogenous C20orf24 level in ECSCs and DNLCs with anti-C20orf24 (i). Band intensities normalized to HSPD1 loading control (LC). t-SNE (t-distributed stochastic neighbor embedding) plot (ii; number of single cells shown in parentheses) and log_2_ UMI (unique molecular identifier) counts (ii; *p value by Wilcoxon rank-sum test) of the C20orf24 transcript in DNLCs over ECSCs. (D) Respirasome activity (fold change, FC) of *C20orf24* KO or complemented with FLAG-tagged C20orf24 (rescue) versus control (ctrl) single-guide RNA (sgRNA) in DNLCs (i). Respirasome complexes in control and *C20orf24* KO DNLCs by BN-PAGE (ii); percent reduction of respirasome complex subunits in KO over control is shown after subtracting background signals from each band. (E) Oxygen consumption rate (OCR) measured in control and KO DNLCs under basal conditions followed by the sequential addition of oligomycin (1 μM), FCCP (0.25 μM; carbonyl cyanide-p-trifluoromethoxyphenylhydrazone), as well as rotenone and antimycin A (0.5 μM). (F) C20orf24 mRNA or respirasome activity measured by qRT-PCR (i; FC, fold change), RNA sequencing (ii), or colorimetric assay (iii) in the CIV-deficient fibroblasts (CDFs) of two subjects (S1, S2) versus healthy fibroblasts (HFs). Immunoblotting (i) of endogenous C20orf24 level in HFs or CDFs (S2) with anti-C20orf24. Band intensities normalized to HSPD1 loading control (LC). (G) C20orf24 mRNA or respirasome activity measured by qRT-PCR or colorimetric assay in *C20orf24* knockdown (KD) versus control (ctrl) of HFs (i) or CDFs of S2 complemented with C20orf24-FLAG (rescue) versus GFP-FLAG control (ii). Data for (D, E, F, and G) are mean ± SD (*n* = 3 technical replicates; *p ≤ 0.05 by Student's t test). See also Figure S5 and Table S1.

The importance of mt respiratory function and energy metabolism for neuronal differentiation motivated us to explore the extent to which orphan proteins with no reported functions in MPCs were enriched for physical links with proteins known to be involved in these processes. When compared with other orphans in static (ECSCs, DNLCs) or DF networks, we observed that the mt orphan C20orf24 displayed a significant (1.8 × 10^−31^ < p < 1.7 × 10^−2^) number of associations with proteins functioning in cellular respiration, generation of precursor metabolites and energy, and metabolic processes in DNLC and DF networks (Figure S5C; Table S3). As well, C20orf24 was found to interact with several known members of the mt respirasome complexes, including CI (10 of 45), CIII (4 of 11), and CIV (7 of 13) in the DNLC network. We confirmed these interactions in NTera2 (ECSCs, DNLCs) and/or in mouse brain lysates by co-IP (Figure 5A). This is further in agreement with the molecular docking analysis (Transparent Methods) that showed the putative interaction interface between the 3D modeled structure of C20orf24 and the solved structure of the human respirasome (Guo et al., 2017), particularly with the subunits of CI (NDUFA3/C1/C2/B9/S5, MTND2/ND6), CIII (UQCR11, UQCRC1, MTCYB), and CIV (COX1/3/6A/7A; Figure 5B). We therefore hypothesized that C20orf24 acts as an assembly factor by interacting with CI/III/IV subunits, where its increased expression during neuronal differentiation in healthy individuals enhances respirasome formation, oxygen consumption, and ATP production (Figure 5A) to protect neurons from energy depletion. Conversely, altered C20orf24 expression during differentiation could result in clinical respiratory chain deficiencies.

Consistent with our hypothesis, C20orf24 levels measured by immunoblotting from the mt extracts of DNLCs were 5-fold higher than in ECSCs, similar to the increase of C20orf24 transcript levels detected in DNLCs by scRNA-seq (Figure 5C). MitoCarta 2.0, an updated inventory of human MPs, predicted C20orf24 to localize to mt (Calvo et al., 2016), whereas amino acid sequence analysis using Phobius (a membrane protein topology method) predicted C20orf24 as having two helical transmembrane domains (Figure S6A), suggesting that it is likely to reside in the IMM. Consistent with these results, we observed the association of C20orf24 in the mitoplasts (IMM and matrix) of mouse brain (or in DNLCs) and in the membrane pellet fraction of mt extracts with NaCl or Na_2_CO_3_, as did IMM, intermembrane space (IMS), and OMM marker proteins (Figure S6B; data for mouse brain shown), indicating membrane localization.

To further establish the subcellular mt localization of C20orf24, we examined mt isolated from mouse brain using proteinase K treatment. If C20orf24 is localized on the IMM, we expect it to be protected from proteinase K digestion. Indeed, in contrast to the OMM marker protein (MFF), we found C20orf24 to be resistant to protease digestion, as were the matrix (HSPD1), IMM (NDUFA4), and IMS (OPA1) marker proteins. Alternately, when the mt was treated with proteinase K in the presence of osmotic shock to selectively open the OMM, C20orf24, like NDUFA4 and HSPD1, was still protected (Figure S6B), whereas OPA1 and MFF were degraded, indicating C20orf24 as an integral IMM protein. Also, the interaction of C20orf24 with the members of the TOMM40 complex (made up of a receptor protein TOMM22, pore-forming subunit TOMM40, and one of the three small TOMM proteins, TOMM5; Figure 3A) further suggests that despite the absence of an mt-targeting signal sequence, C20orf24, like other nuclear-encoded MPs (Kojima et al., 2016), is reliant on translocase activity to reach the IMM.

If C20orf24 expression is elevated in DNLCs and genuinely associated with the subunits of respiratory complexes, its loss of function is likely to affect respirasome activity. To assess this, we created *C20orf24* KOs (Figure S6C) in DNLCs and monitored the stability and activity of respirasome complexes using colorimetric and blue native polyacrylamide gel electrophoresis (BN-PAGE) assays. In both these methods, loss of *C20orf24* in DNLCs consistently reduced respirasome subunit levels (Figure 5D), as well as mt respiratory chain functions (Figure S6D), including the oxygen consumption rate under basal conditions and upon stimulation with FCCP (carbonyl cyanide-p-trifluoromethoxyphenylhydrazone, an uncoupler of OXPHOS; Figure 5E), with no corresponding change in extracellular acidification rate (an indicator of glycolytic activity; Figure S6D). Remarkably, complementation of exogenous FLAG-tagged C20orf24 in the stable *C20orf24* KO DNLCs restored the respirasome levels (Figure 5D), supporting the hypothesis that C20orf24 is a previously unknown assembly factor associated with the respirasome.

Alignment of the C20orf24 protein sequence against the subunits of the respirasome complex showed that C20orf24 shared more than 20% amino acid sequence identity with the CIV subunit COX6C (Figure S6E), whereas the sequence identity dropped below 20% with all other respirasome components. This observation, and the association of C20orf24 with the CIV subunits, inspired us to decipher the role of C20orf24 in CIV deficiency. Although reduced C20orf24 mRNA was observed in CIV-deficient fibroblasts (CDFs) from two unrelated subjects using quantitative real-time PCR (qRT-PCR; Figure 5F), a marked decrease of C20orf24 in the CDFs of one of the subjects led us to examine its level and respirasome activity in this sample using other measures. As with reduced C20orf24 mRNA (or protein), respirasome activity was also significantly (p ≤ 0.05) depleted in this patient's CDFs using RNA-seq and CI/III/IV colorimetric assays (Figure 5F), suggesting that reduced C20orf24 lessens respirasome function.

Next, we performed two assays to determine if the reduction of C20orf24 mRNA can result in the phenotypic characteristics observed in CIV-deficient patient cells (Figure 5G). First, using an small interfering RNA (siRNA)-mediated *C20orf24* knockdown in healthy fibroblasts, we found that reduction in C20orf24 mRNA significantly (p ≤ 0.05) lowered respirasome levels compared with a non-targeting siRNA control. Second, C20orf24 mRNA expression and respirasome activity was fully restored in CDFs expressing wild-type C20orf24, suggesting that C20orf24 plays a key role in maintaining respirasome activity in patients deficient in CIV. Whole-exome sequencing analyses of 295 Japanese patients with childhood-onset mt respiratory chain complex deficiencies, including 55 with defects in CIV, revealed that 29 patients carried 2 homozygous and 12 heterozygous variants in the C20orf24 region: 7 intronic and 5 in the 3′ UTR (Table S1). Existing exome or genome aggregation databases were used to identify the frequency of these alleles in the overall population, and classify them as common (with a minor allelic frequency [MAF] > 5%), less frequent (MAF 1%–5%), or rare (MAF <1%). Among the 14 variants, 16 patients (6 with CI, 1 with CIII, 3 with CIV, 6 with combined respiratory chain complex deficiencies) carried a less frequent heterozygous variant in the 3′ UTR of C20orf24 (GenBank: NM_199483.2 [c.*226G > A]; NM_001199534.1 [c.*402G > A]; NM_018840.4 [c.*398G > A]). We therefore pursued the principal C20orf24 transcript variant (c.*398G > A) for further analysis of its functional effect on respirasome activity.

As 3′ UTRs are a convergence point for gene expression regulation by microRNAs (miRNAs), we hypothesized that in patients with CIV deficiency, miRNAs differentially regulate respirasome levels via C20orf24. To investigate this, we identified two human miRNA species using miRTarbase (hsa-miR-452-3p, -532-3p), binding in regions of the variant c.*398G > A. The miRIDIAN hairpin inhibitors designed specifically to target the two selected miRNAs in treated CDFs (of second subject) increased the C20orf24 mRNA and respirasome activity compared with a non-targeting miRIDIAN miRNA hairpin inhibitor control (Figure S6F). To ascertain if the observed increase of C20orf24 mRNA and respirasome activity in CDFs is due to the 3′ UTR of the C20orf24 variant affected by miRNAs, we ectopically expressed the wild-type (c.398G > G) and variant (c.*398G > A found in the patients with CIV deficiency) 3′ UTR of C20orf24 in healthy fibroblasts downstream of the C20orf24 coding sequence. We found that the variant showed considerable reduction of C20orf24 mRNA and respirasome activity relative to wild-type (Figure S6G). Conversely, the healthy fibroblasts containing the C20orf24 variant significantly (p ≤ 0.05) increased C20orf24 mRNA expression and respirasome activity when treated with a select miRIDIAN hairpin inhibitor (hsa-miR-452-3p), when compared with healthy fibroblasts transfected with wild-type or with variant and control inhibitor (Figure S6G), indicating that the *C20orf24* variant in respirasome function for patients with CIV-deficiency is via miRNA-mediated gene silencing. Jointly, based on its interaction with CI/III/IV subunits and its role in respiratory function, we propose to rename C20orf24 as RCAF1 (respirasome complex assembly factor 1), a candidate gene for respirasome and human respiratory complex chain deficiencies.

### NENF Binding with DJ-1 and PINK1 Is Vital for Neurotrophic Activity in DNLCs

**Figure 6:**
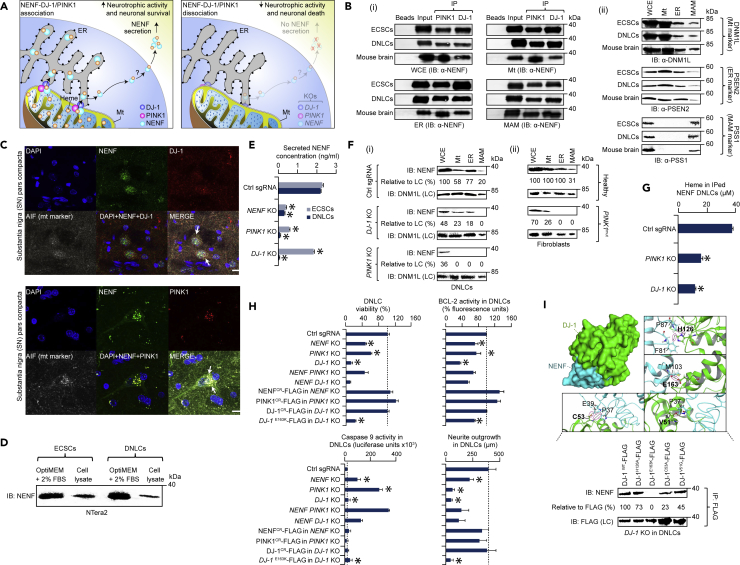
NENF Association with DJ-1 and PINK1 in Neurogenesis (A) Model of NENF role with DJ-1 and PINK1 in neurotrophic activity and neuronal survival/death; question mark indicates the mechanistic pathway in which NENF secreted is unclear. (B) PINK1 and DJ-1 immunoprecipitates in whole-cell extract (WCE) as well as in mt, ER, and MAM lysates from ECSCs, DNLCs, and mouse brain using anti-PINK1 and DJ-1 antibodies immunoblotted (IB) with anti-NENF antibody (i). mt, ER, and MAM markers (ii) used as internal control. (C) Representative confocal micrographs of NENF co-localization (arrow heads) with DJ-1 and PINK1 in the rat SN region using antibodies specific to these native proteins and mt marker (AIF, apoptosis-inducing factor); DNA visualized by DAPI (blue), scale bar, 20 μm. (D) NENF secretion in the conditioned medium and cell lysates immunoblotted (IB) with anti-NENF antibody. (E) Secreted NENF measured in control (ctrl) single-guide RNA (sgRNA) and single KOs of ECSCs or DNLCs. (F) Disruption of NENF association with DJ-1 and PINK1 in the indicated fractions of KOs versus control sgRNA in DNLCs (i) or in the fibroblasts of patients with PD with *PINK1* mutation versus healthy control (ii). Band intensities normalized to loading control (LC). (G) Heme measured in DNLCs of control sgRNA and indicated KOs with NENF immunoprecipitates. (H) Neurotrophic (cell viability, neurite outgrowth) and apoptotic activities (BCL-2, caspase-9) in control versus KOs, CRISPR-resistant (CR) genes fused with FLAG in indicated KOs versus control, or DJ-1 wild-type (WT) versus catalytic (E163K) mutant fused with FLAG in *DJ-1* KO DNLCs. Data in (E, G, and H) are mean ± SD (*n* = 3 biological replicates; *control versus single KOs or DJ-1 WT versus variant (E163K) with p ≤ 0.05 by Student's t test). (I) DJ-1 (PDB ID: 3SF8) with potential interface residues (bold) binding with NENF. Immunoprecipitates of DJ-1 WT or catalytic mutants fused with FLAG in *DJ-1* KO DNLCs using anti-FLAG antibody IB with anti-NENF or FLAG antibody. Band intensities normalized to LC. See also Figure S6.

Another interesting interaction observed in our DNLC network (Figure 6A) was between an NENF protein involved in neuronal differentiation and survival (Kimura et al., 2008) and a PD-causing protein (DJ-1, the product of *PARK7* gene) implicated in the maintenance of mt homeostasis (Ottolini et al., 2013). Although neurotrophic activity of NENF is dependent on heme binding, the mechanism by which heme interacts with NENF still remains unclear (Kimura et al., 2013). Association of NENF with DJ-1 therefore led us to examine its role in binding with heme to promote neurotrophic activity and neuronal survival.

NENF, which is endogenously expressed in mouse brain (Kimura et al., 2008), including in rat or human substantia nigra (SN) and CA1 hippocampal regions (Figures S7A and S7B), contains a secretory signal peptide and mt-targeting sequence (predicted by Phobius, Target P1.1). Immunoblots of subcellular fractions and immunocytochemical analyses of NTera2 cells and mouse or rat brain regions (SN, hippocampal) showed an accumulation of NENF in the mt, endoplasmic reticulum (ER), and mitochondria-associated membrane (MAM) fractions (Figures 6B and S7C; data for SN shown). As with DJ-1 (Ottolini et al., 2013, Zhang et al., 2005), the presence of NENF in the mt and MAM fractions allowed us to further explore if NENF and DJ-1 co-localized between mt and ER. Using co-IP and/or immunocytochemistry in NTera2 (ECSCs, DNLCs), mouse brain, and rat CA1 hippocampal or SN lysates, we found that NENF preferentially binds with DJ-1 in the mt, ER, and MAM fractions (Figures 6B, 6C, and S7D; data for SN shown). Similarly, NENF was found to interact with a PD-contributing protein, PINK1 (PTEN-induced putative kinase 1; Figure 6B), that localizes to MAM (Gelmetti et al., 2017) and physically associates with DJ-1 (Tang et al., 2006). Consistent with this finding, we observed NENF to be associated with DJ-1 and PINK1 in the SN of healthy subjects and those with sporadic PD (Figure S7B), suggesting this binding to occur both in the healthy human brain and in PD.

Based on our observations, we postulated that NENF binding with DJ-1 and PINK1 facilitates the loading of heme from mt, which promotes neuronal survival because of the increased neurotrophic activity of NENF. However, dissociation of the NENF-DJ-1 (or PINK1) interaction might impede the loading of heme into NENF, resulting in neuronal cell death due to reduced neurotrophic activity of NENF (Figure 6A). To investigate this, we attempted to detect endogenous NENF secretion in ECSCs and DNLCs and found that NENF was discernible in conditioned culture medium and cell lysates (Figure 6D), as previously reported in mouse neuroblastoma cells (Kimura et al., 2008). However, as in *NENF* KO, the endogenously secreted *NENF* was significantly (p ≤ 0.05) less prominent in the conditioned medium of ECSCs or DNLCs with *DJ-1* and *PINK1* KOs compared with control cells (Figures 6E and S7E). This finding prompted us to examine if DJ-1 and PINK1 have any underlying role in the localization of NENF in mt, ER, and MAM compartments. We therefore performed immunoblotting using an anti-NENF antibody to detect the endogenous NENF in mt, ER, and MAM extracts of DNLCs with *DJ-1* or *PINK1* KOs, and fibroblasts derived from subjects with PD with a *PINK1* mutation. In contrast to control DNLCs or fibroblasts from healthy subjects, NENF association with DJ-1 and PINK1 was disrupted at different degrees in the mt, ER, and MAM fractions of *DJ-1* and *PINK1* KO cells, in a manner analogous to that seen in the fibroblasts of subjects with PD with a *PINK1* mutation (Figure 6F). The amount of heme in immuniprecipitated NENF was also significantly (p ≤ 0.05) reduced in *DJ-1* and *PINK1* KOs (Figure 6G), when compared with control DNLCs.

To establish if the dissociation of NENF coupling with DJ-1 and PINK1 has any effect on neurotrophic and apoptotic activities, we examined *NENF* and *DJ-1* or *PINK1* single and double KOs in DNLCs. We found that when compared with control DNLCs, the inhibition of endogenous *NENF* significantly (p ≤ 0.05) reduced cell viability, neurite outgrowth, and expression of an anti-apoptotic BCL-2, with increased pro-apoptotic caspase-9 activity, similar to that observed with *DJ-1* or *PINK1* KOs. Exogenous expression of CRISPR-resistant NENF, PINK1, or DJ-1 fully rescued these phenotypic defects in the respective KO lines (Figure 6H). However, inhibition of both *NENF* and *DJ-1* or *PINK1* did not enhance the effects, suggesting functional cooperation between these proteins in the neurotrophic and apoptotic activities of NENF.

We next predicted the amino acid residue sites of the NENF-DJ-1 association using the available crystal structures of these interacting partners in the Protein DataBank. Specifically, we searched for known mutations on residues located at or near the interaction interface of these proteins, finding four (C53, E163, H126, V51) at the DJ-1 interaction interface (Figure 6I). These four mutations have been previously shown to abrogate the protective activity of DJ-1 (Chen et al., 2010, Shendelman et al., 2004, Taira et al., 2004), with one in particular (homozygous mutation in exon 7, E163) having been identified in an Italian family with three brothers affected by early-onset parkinsonism, dementia, and amyotrophic lateral sclerosis (Annesi et al., 2005). Further assessment of these *DJ-1* mutations showed that E163K (of the four mutant *DJ-1* FLAG variants tested) impaired the NENF-DJ-1 association in DNLCs (Figure 6I), resulting in significant (p ≤ 0.05) reduction in neurotrophic and increase in pro-apoptotic (or decrease in anti-apoptotic) activities similar to that observed in *DJ-1* KO cells (Figure 6H). Although the findings open further avenues to explore the mechanistic pathway in which NENF is secreted, our results support a model where NENF-DJ-1-PINK1 association mediates heme loading from mt, which is essential for neurotrophic activity and neuronal survival, whereas perturbations to these interactions result in neuronal death and may be implicated in PD.

## Discussion

Although mapping human cellular interaction networks has continued to reveal important insights into protein function, the full repertoire of cell-context-dependent, native human mtPPIs and phosphorylation sites in MPs that control specific molecular events during neuronal differentiation or how the mtPPI networks change in physiological cell states remains unknown. Our systematic BF/MS approach presented here furthers our understanding of the modular architecture of MPs and the reshuffling of their physical associations as an adaptive response to the physiological cues of neurogenesis.

After rigorously benchmarking and validating mtPPIs from two static (ECSCs, DNLCs) networks using complementary experimental approaches, we identified several unexpected mtPPIs and MPCs annotated to processes critical for neuronal function. Furthermore, the static networks implicated distinct mt hub proteins or interactions for specific cell states, roles for orphan proteins in mt function, subunits of MPCs linked to a spectrum of mt disorders, and CI deficiency-specific perturbations of mtPPIs for a testable disease-relevant outcome. As well, phosphoproteome analysis revealed (1) phosphosites that were not previously reported in the pluripotent state and upon differentiation, (2) distinct phosphosites within the MPCs, (3) cell state-specific mt kinase-substrate pairs, and (4) PDHA2 S291/S293 as an important phosphorylation target that decreased PDHA2 activity in ECSCs, and increased it in DNLCs by dephosphorylation, possibly to support energy requirements for neurons.

Although many identified cell state-dependent PPIs can lend further opportunities to interrogate underlying molecular mechanisms, we focused on two disease-relevant findings from our DNLC network using fibroblasts derived from patients with PD or CIV deficiency. First, the interaction between C20orf24 and CI/III/IV subunits in DNLCs revealed (1) the role of C20orf24 as an assembly factor in stabilizing the activity of respiratory complexes, (2) a relationship between *C20orf24* expression and respirasome levels in DNLCs and healthy fibroblasts, (3) correction of CIV deficiency in patients with reduced respirasome activity through C20orf24 transfection in fibroblasts, (4) a less frequent heterozygous genetic variant c.398G > A in the 3′ UTR of C20orf24 in 16 of the 295 unsolved patients with respiratory chain complex deficiencies, and (5) restoration of respirasome levels by inhibition of two miRNA species targeting the site of c.398G > A in CDFs, suggesting miRNA-mediated regulation of C20orf24 as a contributor to respirasome defects. Although our work provides an in-depth evaluation of C20orf24, much remains to be learned about the contribution of the C20orf24 3′ UTR variant toward respiratory chain deficiencies, and the molecular mechanism by which C20orf24 enhances respirasome formation and activity.

In our second model we observed NENF binding with DJ-1 and PINK1 in the DNLCs and SNs of rat brain or healthy individuals and patients with PD. Abrogation of NENF binding by *PINK1* mutation in the fibroblasts of a patient with PD, or PD-related *DJ-1* variant (E163K) in DNLCs, implicates this interaction in the disease. We also noticed as in *NENF* KOs, reduced heme production, neuronal viability, neurite outgrowth, and anti-apoptotic activities in the absence of *DJ-1* and *PINK1*, providing mechanistic evidence that the association of NENF with DJ-1 and PINK1 is required for localization of NENF at MAMs, and for loading heme from mt to enhance neurotrophic activity and neuronal survival. Beyond establishing the mechanistic role for PPIs of interest, all constructs (Addgene) and data (http://tap.med.utoronto.ca/HsMTProt; PRIDE communal repository: PXD009831-34) have been released as a resource to create avenues for further exploration of MP function in neurogenesis. Through continued examination, we can also identify MPs linked with PD and respiratory chain deficiencies as targets to ameliorate mt dysfunction and consequent neurogenesis-dependent cognitive and metabolic defects.

### Limitations of the Study

As our cross-linker-based BF/MS strategy preferentially detected more stable, higher abundance, and transient mt interactions in native physiological conditions, we may not have captured all relevant human neuronal protein assemblies in the model cell line used. As well, detergent solubilization of the mt membrane may have disrupted interactions with hydrophobic proteins. Mapping interactions of the DNLC network to scRNA-seq indicated that three-fourths (2,455, 69%) of the interacting proteins found to co-elute are primarily from a neuronal cell type, and the remaining are from mixed cell-type populations (Table S2). While these protein associations suggest their requirement for neuronal maintenance and function, their binding within the same or in distinct cell types could be either direct or indirect. Thus the current study could be expanded by performing BF/MS on different cell types at the single-cell level after fluorescence-activated cell sorting from heterogeneous cell populations. Nevertheless, the high-quality mtPPIs we report will be a valuable resource in detailing molecular mechanisms of MPs critical for neurodevelopment, brain function, and mt disease.

## Methods

All methods can be found in the accompanying Transparent Methods supplemental file.

## Supplemental Information

Document S1. Transparent Methods and Figures S1–S7

Table S1. Differentially Expressed Transcripts in DNLCs by Bulk RNA-Seq or scRNA-Seq, and C20orf24 Variants in CIV-Deficient Patient Fibroblasts from Whole-Exome Sequencing, Related to Figures 1, 5, S1, and Transparent Methods

Table S2. Scoring and Validation of High-Quality Protein-Protein Interactions, Related to Figures 2, 3, and Transparent Methods

Table S3. Identification of Putative MPCs and Orphan Associations in the Networks Enriched for Gene Ontology (GO) Processes, Related to Figures 3, S4, S5, and Transparent Methods

Table S4. Phosphorylation Sites and Kinase-Substrate Associations Detected in ECSCs and/or DNLCs, Related to Figure 4 and Transparent Methods
