# Supplementary material for: Rewiring of the Human Mitochondrial Interactome during Neuronal Reprogramming Reveals Regulators of the Respirasome and Neurogenesis
Source: iScience. 2019 Sep 4;19:1114–32. doi: 10.1016/j.isci.2019.08.057 (PMC6831851; doi:10.1016/j.isci.2019.08.057)
Supplement: Document S1. Transparent Methods and Figures S1–S7 [file mmc1.pdf]

ISCI, Volume *19*

Supplemental Information

Rewiring of the Human Mitochondrial Interactome during Neuronal Reprogramming Reveals Regulators of the Respirasome and Neurogenesis

Mohamed Taha Moutaoufik, Ramy Malty, Shahreen Amin, Qingzhou Zhang, Sadhna Phanse, Alla Gagarinova, Mara Zilocchi, Larissa Hoell, Zoran Minic, Maria Gagarinova, Hiroyuki Aoki, Jocelyn Stockwell, Matthew Jessulat, Florian Goebels, Kirsten Broderick, Nichollas E. Scott, James Vlasblom, Gabriel Musso, Bhanu Prasad, Eleonora Lamantea, Barbara Garavaglia, Alex Rajput, Kei Murayama, Yasushi Okazaki, Leonard J. Foster, Gary D. Bader, Francisco S. Cayabyab, and Mohan Babu

SUPPLEMENTARY TABLE LEGENDS

Table S1. Differentially expressed transcripts in DNLCs by bulk RNA-seq or scRNA-seq, and C20orf24 variants in CIV-deficient patient fibroblasts from whole exome sequencing. Related to Figures 1, 5, S1, and Transparent Methods.

Table S2. Scoring and validation of high-quality protein-protein interactions. Related to Figures 2, 3, and Transparent Methods.

Table S3. Identification of putative MPCs and orphan associations in the networks enriched for Gene Ontology (GO) processes. Related to Figures 3, S4, S5, and Transparent Methods.

Table S4. Phosphorylation sites and kinase-substrate associations detected in ECSCs and/or DNLCs. Related to Figure 4 and Transparent Methods.

TRANSPARENT METHODS

Detailed methods are provided below and include the following:

- **KEY RESOURCES TABLE**
- **EXPERIMENTAL MODEL AND SUBJECT DETAILS**
    - Cell Culture and Differentiation
    - Animal Subjects and Tissue Slice Preparation
    - Patient Information
    - Human Skin Fibroblast Cultures
- **METHOD DETAILS**
    - Preparation of Cellular Fractions from Subcellular Components
    - BF/MS
    - Phosphopeptide Enrichment by IMAC and TiO_2
    - Bulk RNA-Sequencing (RNA-Seq) and Single Cell RNA-Sequencing (ScRNA-seq)
    - Immunocytochemistry, Immunohistochemistry, and Confocal Imaging
    - IP and Immunoblotting
    - BN-PAGE and CI/III/IV Colorimetric Assays
    - Oxygen Consumption Rate (OCR) and Extracellular Acidification Rate (ECAR)
    - Structural Modeling and Docking
    - Generation of KOs and Mutagenesis
    - PDH (or PDHA2) Activity and Glutamate Excitotoxicity
    - Quantitative Real-time PCR
    - miRNA or siRNA Transfection

- Quantification of Cellular Heme and NENF Secretion
- Neurotrophic and Apoptotic Assays

- **QUANTIFICATION AND STATISTICAL ANALYSIS**
  - Data Filtering, Scoring mtPPIs, and Defining MPCs
  - Bulk RNA-seq and ScRNA-seq
  - Identification of High-Confidence Phosphosites
  - Enrichment Analyses
  - Kinase-Substrate Network
  - Statistical Tests

- **DATA AND SOFTWARE AVAILABILITY**

KEY RESOURCES TABLE

REAGENT or RESOURCE	SOURCE	IDENTIFIER
**Antibodies**		
Anti-DJ-1	Protein Tech, Abcam	Cat# 11681-1-AP, ab119767
Anti-PINK1	Abcam, Santa Cruz, Invitrogen	Cat# ab23707, sc-517353, PIPA518770
Anti-NENF	Abcam	Cat# ab74474
Anti-C20orf24	LsBio	Cat# LS-C81366
Anti-NDUFA4	Abcam	Cat# ab129752
Anti-NDUFS3	Abcam	Cat# ab177471
Anti-NDUFV1	Abcam	Cat# ab55535
Anti-NDUFA9	Abcam	Cat# ab181381
Anti-NDUFS8	Abcam	Cat# ab170936
Anti-UQCRC2	Abcam	Cat# ab103616
Anti-COX5A	Santa Cruz	Cat# sc376907
Anti-COX7C	Abcam	Cat# ab150953
Anti-GAPDH	Abcam	Cat# ab9485
Anti-NIPSNAP1	Santa Cruz	Cat# sc-393201
Anti-TOMM22	Santa Cruz	Cat# sc-58308
Anti-TOMM40	Abcam	Cat# ab51884
Anti-MTCH2	Abcam	Cat# ab174921
Anti-TOMM5	Abcam	Cat# ab151037
Anti-NDUFS1	Abcam	Cat# ab169540
Anti-PSEN1	Abcam	Cat# ab76083
Anti-SLC1A3	Abcam	Cat# ab416

Anti-SIRT3	Novus Bio	Cat# MAB7488
Anti-PRKACA	Cell Signaling	Cat# 2056S
Anti-GJA1	Novus Bio	Cat# MAB7737
Anti-ATP2A2	Abcam	Cat# ab2861
Anti-pS	Abcam	Cat# ab9332
Anti-pT	Abcam	Cat# ab9337
Anti-PDHA2	Novus Bio	Cat# NBP1-79536
Anti-PDHA2 (pS293)	Novus Bio	Cat#NB11093479
Anti-BCL2	Abcam, Santa Cruz	Cat# ab32124, sc-7382
Anti-DNM1L	Abcam	Cat# ab56788
Anti-PSEN2	Abcam	Cat# ab51249
Anti-PSS1 (PTDSS1)	Santa Cruz	Cat# sc-515376
Anti-FLAG	Sigma	Cat# F1804
Anti-HSPD1	Abcam	Cat# ab46798
Anti-TUBB3	Santa Cruz	Cat# sc-80005
Anti-NEFL	Santa Cruz	Cat# sc-20012
Anti-NEUN	Abcam	Cat# ab177487
Anti-VIM	Santa Cruz	Cat# sc-6260
Anti-ANXA1	Santa Cruz	Cat# sc-12740
Anti-CRMP5 (DPYSL5)	Santa Cruz	Cat# sc-58515
Anti-NRF1	Santa Cruz	Cat# sc-515360
Anti-PSAP	Santa Cruz	Cat# sc-390184
Anti-MFF	Abcam	Cat# ab81127
Anti-OPA1	Santa Cruz	Cat# sc-393296
Anti-SOX2	Santa Cruz	Cat# sc-365964
Anti-TAU	Santa Cruz	Cat #sc-32274
Anti-MAP2	Santa Cruz	Cat #sc-74421
Goat anti-rabbit IgG (H+L) secondary Ab, Alexa Fluor 488 for anti-BCL2	Abcam	Cat# ab15077
Goat anti-mouse IgG (H+L) secondary Ab, Alexa Fluor 488 for anti-TUBB3	Abcam	Cat# ab9871
Goat anti-rabbit IgG (H+L) secondary Ab, Alexa Fluor 488	Thermo Fisher Scientific	Cat# A-11034
Secondary HRP anti-rabbit	Sigma	Cat# A6154
Secondary HRP anti-mouse	Sigma	Cat# A4416
Anti-AIF (Alexa Fluor 647)	Abcam	Cat# ab196847
Anti-KDEL (Alexa Fluor 555)	Abcam	Cat# ab203420
Goat anti-rabbit IgG (H+L) Alexa Fluor 488, polyclonal, secondary Ab	Thermo Fisher Scientific	Cat# A-11034
Goat anti-rabbit IgG (H+L) Alexa Fluor 647, polyclonal, secondary Ab	Thermo Fisher Scientific	Cat# A-21244
Donkey anti-Rabbit IgG (H+L), Alexa Fluor 488, polyclonal, secondary Ab	Thermo Fisher Scientific	Cat# A-21206

Goat anti-mouse IgG (H+L) Alexa Fluor 488, polyclonal, secondary Ab	Thermo Fisher Scientific	Cat# A-11001
Donkey anti-mouse IgG (H+L), Alexa Fluor 568, polyclonal, secondary Ab	Thermo Fisher Scientific	Cat# A-10037
Donkey anti-goat IgG (H+L) Alexa Fluor 488, polyclonal, secondary Ab	Thermo Fisher Scientific	Cat# A-11055
Rabbit anti-goat IgG (H+L) Alexa Fluor 546, Polyclonal, Secondary Ab	Thermo Fisher Scientific	Cat# A-21085
**Bacterial and Virus Strains**		
DH5α competent cells	Thermo Fisher Scientific	Cat# 18255017
SURE 2 competent cells	Agilent Technologies	Cat #200152
E. coli NEB stable	New England Biolabs	Cat# C3040I
**Biological Samples**		
Mutant *PINK1* fibroblasts; subject 1 (code: F-PK386)	C. Besta, IRCCS Foundation Neurological Institute, Italy	Male with early-onset PD; homozygous mutation for c.502G>C (p.Ala168Pro); age at biopsy- 59 yrs (biopsy date: June 2003)
Control fibroblasts; subject 1 (code: F-HA498)	C. Besta, IRCCS Foundation Neurological Institute, Italy	Mutation: none; Male; age at biopsy- 54 yrs (biopsy date: Sept 2015); Healthy
Control fibroblasts; subject 2 (code: F-HA499)	C. Besta, IRCCS Foundation Neurological Institute	Mutation: none; Female; age at biopsy- 51 yrs (biopsy date: Sept 2015); Healthy
Mutant NDUFS1 fibroblasts; subject 1 (code: F-DV356)	C. Besta, IRCCS Foundation Neurological Institute, Italy	Female with CI deficiency; compound heterozygous NDUFS1 mutations: c.2083T>C/p.Tyr695His and c.2084A>G/p.Tyr695Cys; age at biopsy- 1 yr (biopsy date: Nov 2006)
Mutant NDUFV1 fibroblasts; subject 1 (code: F-MT5869)	C. Besta, IRCCS Foundation Neurological Institute, Italy	Male with CI deficiency; homozygous NDUFV1 mutation c.1268C>T/p.Thr423Met; age at biopsy- 5 yrs (biopsy date: Feb 2012)
Control fibroblasts; subject 1 (code: F- MT8796)	C. Besta, IRCCS Foundation Neurological Institute, Italy	Mutation: none; Female; age at biopsy- 50 yrs (biopsy date: April 2017); Healthy
Control fibroblasts; subject 2 (code: F- MT8534)	C. Besta, IRCCS Foundation Neurological Institute, Italy	Mutation: none; Male; age at biopsy-57 yrs (biopsy date: Oct 2016); Healthy
Human fibroblasts from CIV-deficient patients (Codes: F-MT2979; F-MT6119)	C. Besta, IRCCS Foundation Neurological Institute, Italy	CIV deficiency; F-MT2979 is a homozygous mutation for c.792_793delAG (subject 1), while F-MT6119 (subject 2) is a compound heterozygous mutation for c.588+1G>A and c.751C>T (p.Gln251Ter).

Patients with CI, CIV and combined respiratory chain complex deficiencies	Saitama Medical University Hospital and Chiba Children's Hospital, Japan	Patient codes, gender, age of onset, enzymatic deficiency, as well as clinical diagnosis and presentation are shown in Table S1 (Sheet 2).
Substantia nigra (SN) from healthy control (SK00237) and PD patient (SK00344)	Saskatoon Health Region, Bioethics Board University of Saskatchewan, Canada	Mutation none; after autopsy, one-half of the brain (SN was isolated when needed) from healthy or PD patient was frozen at -80°C, while the other half was fixed in formalin and examined by a neuropathologist.
**Chemicals, Peptides, and Recombinant Proteins**		
SCF (C-Kit Ligand) recombinant protein	Thermo Fisher Scientific	Cat# PHC2111
IL3 recombinant protein	Thermo Fisher Scientific	Cat# PHC0035
GM-CSF recombinant protein	Thermo Fisher Scientific	Cat # PHC2015
Basic fibroblast growth factor	Thermo Fisher Scientific	Cat# PHG0261
6-hydroxydopamine hydrobromide	Sigma-Aldrich	Cat# 162957-50MG
DSP	Thermo Fisher Scientific	Cat# 22585
DMEM	GE Healthcare	Cat# SH30243.LS
FBS	Thermo Fisher Scientific	Cat# 12483020
Penicillin/Streptomycin	Thermo Fisher Scientific	Cat# SV30010
Magnetic beads/columns	Miltenyi Biotec GmbH	Cat# 130-042-601, 130-042-701
Trypsin	Promega	Cat# V5280
Opti-MEM I	Thermo Fisher Scientific	Cat# 31985088
Lipofectamine LTX	Thermo Fisher Scientific	Cat# 15338100
Lipofectamine 2000	Thermo Fisher Scientific	Cat# 11668019
Lipofectamine RNAiMAX	Thermo Fisher Scientific	Cat# 13778075
Retinoic acid	Sigma-Aldrich	Cat# R2625
Hexadimethrine bromide	Sigma-Aldrich	Cat# H9268
Digitonin	GoldBio	Cat# D-180-2.5
Protease/phosphatase inhibitors	EMD Millipore	Cat# 539133, 524628
Coenzyme A	Calbiochem	Cat# 234101
Adenosine 5 Triphosphate disodium salt	Calbiochem	Cat# 1191
NAD, Free acid, Grade I	Roche Diagnostic GmBH	Cat # 10127965001
ß-glycerol phosphate disodium salt	Sigma	Cat # 50020
Rotenone	Sigma	Cat# R8875
Antimycin A	Sigma	Cat# A8674

Carbonyl cyanide 3-chlorophenylhydrazone	Sigma	Cat# C2759
Sodium Pyruvate	Sigma	Cat# P2256
Thiamine pyrophosphate	Sigma	Cat# C8754
Potassium cyanide	Sigma	Cat# 60178
Anti-FLAG M2 beads	Sigma	Cat# F3165
Protein A and Protein G microbeads	Milteny Biotec GmbH	Cat# 130-071-001, 130-071-101
DAPI	Sigma Aldrich	Cat# F6057
Hoechst 33342	Thermo Fisher Scientific	Cat# H3570
MitoTracker	Thermo Fisher Scientific	Cat# M7512
EasySep™ Dead Cell Removal (Annexin V) kit	Stemcell Technologies	Cat# 17899
MK-801	Santa Cruz	Cat# sc-203137
pCR-BluntII-TOPO-PDHA2	Dana Farber	HsCD00347415
pENTR223-DJ-1	Dana Farber	HsCD00383508
pPHAGE-C20orf24-C-TAP	Dana Farber	HsCD00448817
pPHAGE-PINK1-C-TAP	Dana Farber	HsCD00460786
pPHAGE-NENF-C-TAP	Dana Farber	HsCD00458563
pPHAGE-CMV-eGFP-W	Dana Farber	EvNO00061634
**Critical Commercial Assays**		
PDH enzyme activity microplate assay kit	Abcam	Cat# 109902
MTT (3-(4, 5-dimethylthiazolyl-2)-2, 5-diphenyltetrazolium bromide cell proliferation kit	ATCC	Cat# 30-1010K
Pierce Fe-NTA or TiO_2 phosphopeptide kits	Thermo Fisher Scientific	Cat# A32992, A32993
Hs-miR-532-3p-1 miScript primer assay	Qiagen	MS00010052
Hs-miR-452*-2 miScript primer assay	Qiagen	MS00009730
Hs-SNORD68-11 miScript primer assay	Qiagen	MS00033712
RNeasy mini kit	Qiagen	Cat# 74104
Caspase-Glo 9 assay	Promega	Cat# 8210
Human NENF ELISA kit	G-Biosciences	Cat# IT4707
Heme assay kit	Sigma	Cat# MAK316
Mitocheck complex 1 kit	Cayman Chemicals	Cat#700930
Mitocheck complex III kit	Cayman chemicals	Cat #700959
Mitocheck complex IV kit	Cayman Chemicals	Cat #700990
M-MLV reverse transcriptase	Thermo Fisher	Cat#28025-013
Luna Universal qPCR mix	New England Biolabs	M3003S
**Deposited Data**		
Experimental PPIs	BioGRID	PMID: 27980099
Experimental PPIs	HumanNet	PMID: 21536720
Protein complexes	CORUM database	PMID: 17965090
Functional PPIs	STRING	PMID: 27924014

Experimental PPIs	GeneMANIA	PMID: 20576703
Experimental PPIs	AP/MS, BF/MS and XL/MS	PMIDs: 26496610, 28514442, 27499296, 28130547, 29128334, 22939629, 29222160, 26344197
Protein localization and domain annotation	Uniprot, HPA	PMIDs: 27899622, 28495876
Protein abundance	Mouse Brain	PMID: 26523646
mRNA expression	Brain cell types	GEO: GSE52564
Disease ontology ID	Biological and clinical human disease-related data	PMID: 26093607
Mt protein assignment	MitoCarta 2.0, Malty et al	PMIDs: 26450961, 25367773
Disease annotation	DisGenNET	PMID: 27924018
Known phosphorylation sites	PhosphoSitePlus	PMID: 22135298
mRNA expression	Brain cell types	GEO: GSE52564
Protein abundance (LFQ)	Mouse Brain	PMID: 26523646
Transcriptome data of brain development	CORTECON	PMID: 24991954
Disease database	OMIM	PMID: 15608251
Disease database	HGMD	PMID: 24077912
Phenotypes and disease models	MGI	PMID: 29092072
**Experimental Models: Cell Lines**		
NTera2 cl.D1	ATCC	Cat# CRL-1973
SH-SY5Y	ECACC	Cat# 94030304
HEK293T	ATCC	Cat# CRL-3216
**Experimental Models: Organisms/Strains**		
Mus musculus (C57BL/6)	Jackson laboratory	Cat# 000664
Female Wistar rats	Charles river laboratory	Strain code: 003
**Oligonucleotides**		
sgRNA-Control-FOR	Sigma-Millipore	CACCGACGGAGGCTAAGCGTCGCAA
sgRNA-Control-REV	Sigma-Millipore	AAACTTGCGACGCTTAGCCTCCGTC
sgRNA-non targeting control	Sigma-Millipore	ACGGAGGCTAAGCGTCGCAA
sgRNA-PDHA2-FOR	Sigma-Millipore	CACCGATGTAATGACGTGATCCGAG
sgRNA-PDHA2-REV	Sigma-Millipore	AAACCTCGGATCACGTCATTACATC
sgRNA-NENF-FOR	Sigma-Millipore	CACCGACTACAGAGTTTTATGGACG
sgRNA-NENF-REV	Sigma-Millipore	AAACCGTCCATAAAACTCTGTAGTC
sgRNA-DJ-1-FOR	Sigma-Millipore	CACCGAGTACAGTGTAGCCGTGATG
sgRNA-DJ-1-REV	Sigma-Millipore	AAACCATCACGGCTACACTGTACTC
sgRNA-PINK1-FOR	Sigma-Millipore	CACCGCGCCACCATGGCGGTGCGAC
sgRNA-PINK1-REV	Sigma-Millipore	AAACGTCGCACCGCCATGGTGGCGC
sgRNA-PARK2-FOR	Sigma-Millipore	CACCGTCCGACTATTTGTTGCGATC
sgRNA-PARK2-REV	Sigma-Millipore	AAACGATCGCAACAAATAGTCGGAC
sgRNA-C20orf24-FOR	Sigma-Millipore	CACCGTGGACCGCGAATCAGTGTGT
sgRNA- C20orf24-REV	Sigma-Millipore	AAACACACACTGATTCGCGGTCCA
PDHA2-FOR	Sigma-Millipore	TAAGCAGCTAGCATGCTGGCCGCCTTC
PDHA2-REV	Sigma-Millipore	TAAGCAACTAGTTTAACTGACGGACTTAA ACTTGATCC

PDHA2-CR-FOR	Sigma-Millipore	GTACAAACAGAAATTCATTCGCGGTTTCT GTCACCTGTGCGATGGTCAGGAAGCTTG TTGCGTGGGCCTTGAGGCCGGCATAAAC CCCTCGGATCAC
PDHA2-CR-REV	Sigma-Millipore	GTGATCCGAGGGGTTTATGCCGGCCTCA AGGCCCACGCAACAAGCTTCCTGACCAT CGCACAGGTGACAGAAACCGCGAATGAA TTTCTGTTT
PDHA2-mut1	Sigma-Millipore	ATGGAGCTGCAAACCTACCG TT ATCAT GGACAC
PDHA2-mut2	Sigma-Millipore	CCATGATAACGGTAGGTTTGCAGCTCCAT CAGT
PDHA2-mut3	Sigma-Millipore	CGAGAAGAAATTCAGGAAGTAAGAAGTAA GAGG
PDHA2-mut4	Sigma-Millipore	GATCCCTCTTACTTCTTACTTCCTGAATTT CTT
PDHA2-mut-S291D-FOR	Sigma-Millipore	GACATGAGTGATCCTGGAGTCAG TTATCGTACA
PDHA2-mut-S291D-REV	Sigma-Millipore	CTCGTGTACGATAACTGACTCCAGGATCA CTCATGTCGTGT
PDHA2-mut-S293D-FOR	Sigma-Millipore	AGTATGGACGATCCTGGAGTCAG TTATCGTACA
PDHA2-mut-S293D-REV	Sigma-Millipore	CTCGTGTACGATAACTGACTCCAGGATCG TCCATACTGTGT
PDHA2-mut-S291A-FOR	Sigma-Millipore	GCCATGAGTGATCCTGGAGTCAG TTATCGTACA
PDHA2-mut-S291A-REV	Sigma-Millipore	CTCGTGTACGATAACTGACTCCAGGATCA CTCATGGCGTGT
PDHA2-mut-S293A-FOR	Sigma-Millipore	AGTATGGCCGATCCTGGAGTCAG TTATCGTACA
PDHA2-mut-S293A-REV	Sigma-Millipore	CTCGTGTACGATAACTGACTCCAGGATCG GCCATACTGTGT
DJ-1-outer-FOR	Sigma-Millipore	CTGGTCATCCTGGCTAAAGG
DJ-1-outer-REV	Sigma-Millipore	TGTCCTGGCTGGTCTAGAGG
DJ-1-V51G-FOR	Sigma-Millipore	GTGATGTGGGCATTTGTCCTGATG
DJ-1-V51G-REV	Sigma-Millipore	ACAAATGCCCACATCACGGCTAC
DJ-1-C53A-FOR	Sigma-Millipore	GTGGTCATTGCCCCTGATGCCAGC
DJ-1-C53A-REV	Sigma-Millipore	AGGGGCAATGACCACATCACGGC
DJ-1-H126A-FOR	Sigma-Millipore	GTAAAGTTACAACAGCCCCTCTTGCTAAA GAC
DJ-1-H126A-REV	Sigma-Millipore	GCAAGAGGGGCTGTTGTAACTTTACTTCC
DJ-1-E163K-FOR	Sigma-Millipore	ACCAGCTTCAAGTTTGCGCTTG
DJ-1-E163K-REV	Sigma-Millipore	CGCAAACTTGAAGCTGGTCC
DJ-1-FOR	Sigma-Millipore	CAAAATCAACGGGACTTTCC
DJ-1-REV	Sigma-Millipore	CAGGTTGAACAGCTCTCTGG
CR-DJ-1-FOR	Sigma-Millipore	GTGATGTAGTCATTTGTCCTGATGCC
CR-DJ-1-REV	Sigma-Millipore	AATGACTACATCACGGCTACAC
DJ-1 V51G-CR-FOR	Sigma-Millipore	GTGATGTAGGCATTTGTCCTGATGC
DJ-1 V51G-CR-REV	Sigma-Millipore	TGCCTACATCACGGC
DJ-1 C53A-CR-FOR	Sigma-Millipore	GTGATGTAGTCATTGCCCCTGATG
SpeI-EF1a_F	Sigma-Millipore	ATTACAGGGACAGCAGAGATCCAGTTTG GAGGCTCCGGTGCCCGTCAG

NcoI_EF1a_R	Sigma-Millipore	CCTTTCTTTATGTTTTTGGCGTCTTCCATGTCACGACACCTGAAATGGAAGAAAAAAC
C20orf24-3UTR_F	Sigma-Millipore	GGATCATCTTTTACACTGCCATC
C20orf24-3UTR_R	Sigma-Millipore	ACTGGTTCCCAAGCTACAGG
BamHI-3UTR_F	Sigma-Millipore	CCAAGAAGGGCGGAAAGATCGCCGTGTAAGTGGTGTACAGCTCCCAAGTGC
BamHI-3UTR_R	Sigma-Millipore	GGCGCCAAAACCCGGCGCGGAGGCCGGATCGGCTTTATCAGGTATCATCAACAGGTTCTC
C20orf24-outer_F	Sigma-Millipore	GCACAGGAACTTGATCGTTG
C20orf24-outer_R	Sigma-Millipore	GGGCGGAAGGATCAGGAC
C20orf24-inner_F	Sigma-Millipore	AAACAACATGGCTGTATGTGC
C20orf24-inner_R	Sigma-Millipore	CATGTTGTTTCAGAAGACTTGAAATGC
C20orf24-3'UTR-transf_F	Sigma-Millipore	TACCCCTACGACGTGCCCGACTACGCCTAGTGGTGTACAGCTCCCAAGTGC
C20orf24-3'UTR-transf_R	Sigma-Millipore	GGGGGGGGGGCGGAATTTTCTCAATTAAAGATTTGATTTATTCAAGTATGTGAAAACATTC
PINK1-outer_F	Sigma-Millipore	ATCCACGCTGTTTTGACCTC
PINK1-outer_R	Sigma-Millipore	AAGAAGCGGAGACGGTTAGG
PINK1-inner_F	Sigma-Millipore	GACAAGCGCTGGGCCGC
PINK1-inner_R	Sigma-Millipore	CGCTTGTCGCACCGCC
miRIDIAN miRNA human hsa-miR-532-3p -hairpin inhibitor	Dharmacon	IH-301109-02-0002
miRIDIAN miRNA human hsa-miR-452-3p -hairpin inhibitor	Dharmacon	IH-300736-08-0002
miRIDIAN miRNA hairpin inhibitor transfection control with Dy547	Dharmacon	Cat # CP-004500-01-05
siGENOME human C20orf24 siRNA	Dharmacon	Cat # 55969
siGENOME non-targeting siRNA	Dharmacon	Cat # D-001206-13-05
**Recombinant DNA**		
pLEX307_PDHA2-WT	This study	Addgene #115192
pLEX307_PDHA2-S291A	This study	Addgene #115196
pLEX307_PDHA2-S293A	This study	Addgene #115197
pLEX307_PDHA2-S291A S293A	This study	Addgene #115198
pLEX307_CR-PDHA2	This study	Addgene #115199
pLEX307_CR-PDHA2-S291A	This study	Addgene #115203
pLEX307_CR-PDHA2-S293A	This study	Addgene #115204

pLEX307_CR-PDHA2-S291A S293A	Thus study	Addgene #115205
pENTR223-DJ-1-V51G	This study	Addgene #115178
pENTR223-DJ-1-C53A	This study	Addgene #115179
pENTR223-DJ-1-H126A	This study	Addgene #115180
pENTR223-DJ-1E163K	This study	Addgene #115181
pLD-Cc-puro-DJ-1-WT-VA	This study	Addgene #115182
pLD-Cc-puro-DJ-1-V51G-VA	This study	Addgene #115183
pLD-Cc-puro-DJ-1-C53A-VA	This study	Addgene #115184
pLD-Cc-puro-DJ-1-H126A-VA	This study	Addgene #115185
pLD-Cc-puro-DJ-1-E163K-VA	This study	Addgene #115186
pLD-puro-Cc-CR-DJ-1-WT-VA	This study	Addgene # 115187
pLD-puro-Cc-CR-DJ-1-V51G-VA	This study	Addgene #115188
pLD-puro-Cc-CR-DJ-1-C53A-VA	This study	Addgene #115189
pLD-puro-Cc-CR-DJ-1-H126A-VA	This study	Addgene #115190
pLD-puro-Cc-CR-DJ-1-E163K-VA	This study	Addgene # 115191
pLD-puro-CcVA	Moffat Laboratory	Addgene #24588
pPHAGE-C20orf24-C20orf24-3'UTR-WT	This study	Addgene # 128508
pPHAGE-C20orf24-C20orf24-3'UTR-VAR	This study	Addgene # 128509
pPHAGE-CR-PINK1-C-TAP	This study	Addgene # 128510
pPHAGE-CR-NENF-C-TAP	This study	Addgene # 128511
**Software and Algorithms**		
I-TASSER	Protein structure and function prediction	PMID: 25549265
Kallisto	RNA-seq quantification	PMID: 27043002
PIPER	Schrödinger, LLC	Protein-protein docking
Ascore	Probability score	http://ascore.med.harvard.edu/download.php
SEQUEST	Search engine	PMID: 24226387; In house
STATQUEST	Peptide assignment	PMID: 12644571; In house
Comet	Search engine	PMID: 26115965 Version 2016.01 rev. 0
MS-GF+	Search engine	PMID: 25358478 Version 43
X! Tandem	Search engine	PMID: 14976030 Version: 2007.07.01.3
MSblender	Integration of multiple search engine results	PMID: 21488652
DESeq2	R function	Stats: R package
GO sematic similarity	R package	PMID: 20179076
Student's *t*-test	R function	Stats: R package
HiSeq analysis (ver. 2-2.5.55.1311)	Variant calling	Illumina
GPS 3.0 software	Kinase-specific phosphosite	http://gps.biocuckoo.org/download.php

CoreMethod	MATLAB code	PMID: 19193141
ClusterONE	Cytoscape app	PMID: 22426491
Gene Set Enrichment Analysis (GSEA)	GSEA (using g:Profiler)	PMID: 16199517
Hypergeometric test	R function	Stats: R package
Fishers exact test	R function	Stats: R package
Wilcoxon signed-rank test.	R function	Stats: R package
Student's *t*-test	R function	Stats: R package
glmQLFTest	R function	Stats: R package
Network visualization	Cytoscape ver. 3.5.1	PMID: 14597658
miRTarbase	miRNA identification	PMID: 29126174
Band quantification and Neurite outgrowth	ImageJ plugin	PMIDs: 26153368, 22743772; FIJI plugin win64
Four-parameter logistic curve	Quantification of NENF secretion	http://www.myassays.com/four-parameter-logistic-curve.assay
Cell Ranger ver. 3.0.2	10x Genomics	Single cell software downloaded from 10x genomics
Scater ver. 1.12.0	Bioconductor	Stats: R package
Scran ver. 1.12.0	Bioconductor	Stats: R package
DropletUtils ver. 1.4.0	Bioconductor	Stats: R package
Igraph ver. 1.2.4.1	Bioconductor	Stats: R package
**Other**		
Proteomics data deposition	PRIDE	PXD009831-34
Single cell and bulk RNA-seq data (ECSCs, DNLCs)	NCBI	PRJNA474206

EXPERIMENTAL MODEL AND SUBJECT DETAILS

Cell Culture and Differentiation

NTera2 ECSCs were grown under standard conditions (37°C, 5% CO_2) in DMEM medium (Dulbecco's Modified Eagle's Medium) containing 4 mM L-glutamine, 1 mM sodium pyruvate, 10% fetal bovine serum (FBS), penicillin (100 U/mL), and streptomycin (100 µg/mL). The harvested ECSCs were differentiated into neuron-like cells by seeding at 30% confluency in DMEM containing 10 µM retinoic acid (RA), and changing the medium daily for 4 weeks. After confirming the morphological characteristics of ECSCs and DNLCs using an EVOS cell imaging system, harvested cells were cross-linked as with SH-SY5Y neuronal cells (Malty et al., 2017).

Animal Subjects and Tissue Slice Preparation

All experimental procedures were carried out in accordance to guidelines of the Canadian Council for Animal Care under the supervision of the University of Saskatchewan Animal Care and Supply Committee as well as the University of Regina President's Committee for Animal Care. Female Wistar rats and adult C57BL/6 mice were housed in the pathogen-free laboratory animal care-certified barrier facility maintained at 20-24°C with a natural 12 h light-dark cycle. All animals had unlimited access to standard pelleted diet and tap water.

Female Wistar rats were anaesthetized at 12-14 weeks with halothane and rapidly decapitated, with the brains immediately excised and submerged in oxygenated, ice-cold high-sucrose dissection medium (87 mM NaCl, 25 mM $NaHCO_3$, 25 mM glucose, 75 mM sucrose, 2.5 mM KCl, 1.25 mM NaH_2PO4, 7.0 mM $MgCl_2$, 0.5 mM CaCl2). Hippocampal slices taken at 400 µm thickness using a vibrating tissue slicer (Vibram Instruments) was processed in the dissection medium as stated above. Likewise, mice were euthanized by cervical dislocation and brains were quickly removed and washed twice with ice-cold phosphate buffered saline (PBS) containing protease inhibitor cocktail (PIC) and flash-frozen in liquid nitrogen for later use.

Patient Information

Human skin biopsies from healthy and patients harboring the complex I (CI; *NDUFS1/VI*) or Parkinson's disease (PD; *PINK1*) mutations, and deficient for complex IV (CIV) were obtained from IRCCS Foundation Neurological Institute 'C. Besta' (Italy). Likewise, clinically affected tissues (skeletal muscle, liver, heart) or fibroblasts from 295 patients with childhood-onset and mt respiratory chain complex deficiencies were obtained from the Saitama Medical University Hospital and Chiba Children's Hospital (Japan). In all cases, informed written consent from family members was obtained prior to participation in the study, and approval was sought by the

institutional ethics committee. Consent for autopsy is approved by the Saskatoon Health Region and use of autopsy brain for PD research is approved by the University of Saskatchewan Bioethics Board. Autopsy consent is granted by the next-of-kin. Immediately after autopsy, one-half of the brain was frozen at -80°C, while the other half was fixed in formalin and examined by a neuropathologist. Autopsy is performed within 24 h of death.

Human Skin Fibroblast Cultures

Primary skin fibroblasts from healthy and patients with mt respiratory chain complex deficiencies or CI (*NDUFS1*, *VI*) or PD (*PINK1*) mutations were grown as we previously described (Kohda et al., 2016; Malty et al., 2017). Fibroblasts from healthy (F-HA499) and CIV-deficient patients (F-MT6119, F-MT2979) were cultured in DMEM, supplemented with 15% fetal bovine serum (FBS), 1% penicillin/streptomycin, and 1% L-glutamine (Euroclone). Fibroblast cells maintained at 37°C with 5% CO_2 were detached with trypsin, centrifuged at 500 *x*g for 10 min at 25 °C, and then harvested at 80-90% confluency.

METHOD DETAILS

Preparation of Cellular Fractions from Subcellular Components

Mitochondria (Mt), endoplasmic reticulum (ER), and mitochondria-associated membrane (MAM) fractions from NTera2 (ECSCs, DNLCs), mouse brain, and healthy or PD patient fibroblasts with *PINK1* mutation were isolated as outlined previously (Wieckowski et al., 2009; Williamson et al., 2015). Isolation of mitoplasts and OMM, carbonate or NaCl extraction, osmotic shock and proteinase K treatment were performed as described (Perciavalle et al., 2012).

BF/MS

A total of 200 µg mt extracts isolated from NTera2 (ECSCs, DNLCs) was subjected to SEC and IEC techniques using an Agilent 1100 HPLC system with 700 fractions collected. SEC was carried out with a 300 × 7.8 mm BioSep4000 Column (Phenomenex) as per our recently published protocol (Babu et al., 2018), with 84 fractions collected. IEC was performed using a PolyWAX LP column (200 x 4.6 mm id, 5 µm, 1000Å), equilibrating 5 min with buffer A (20 mM Tris-HCl pH 7.8, 0.015% Triton X-100, 1% glycerol) and 30 min linear gradient from 0 to 100% buffer B (Buffer A with 0.6 M NaCl), which resulted in the elution of 87 and 95 fractions in DNLCs and ECSCs, respectively. The fractions were digested with trypsin and analyzed by an Easy-nanoflow liquid chromatography 1000 (Easy nLC; Proxeon) system coupled to an Orbitrap Elite mass spectrometer (ThermoFisher Scientific). Detailed procedures for processing digested samples, chromatographic separation, and control settings for full scanning of mass spectrometry (MS) spectra acquisition are described in our earlier study (Malty et al., 2017).

Phosphopeptide Enrichment by IMAC and TiO_2

Phosphopeptide enrichment by IMAC and TiO_2 was performed as per the protocol from Pierce Fe-NTA or TiO_2 phosphopeptide enrichment kits (ThermoFisher Scientific). Enriched phosphopeptides were analyzed in triplicate by MS on an Orbitrap Elite mass spectrometer using the procedure described (Malty et al., 2017). All samples were acidified with 1% trifluoroacetic acid, desalted (TopTip C-18 columns) and speed-vacuum dried.

Bulk RNA-Sequencing (RNA-Seq) and Single Cell RNA-Sequencing (ScRNA-seq)

Bulk RNA-sequencing was conducted on the NTera2 (ECSCs, DNLCs) cell line, as well as from the fibroblast of a CIV-deficient patient (F-MT6119) and healthy individual (F-HA499) in triplicate at Network Biology Collaborative Centre located at the Lunenfeld-Tanenbaum

Research Institute in Mount Sinai Hospital, or at TCAG (The Centre for Applied Genomics) Toronto Hospital for Sick Children sequencing facility. RNA library was prepared essentially following the protocol of Illumina's NEBNext Ultra II Directional RNA Library Preparation kit.

In brief, double-stranded cDNA synthesized from 800 ng of DNAse-I treated total RNA that was extracted from the indicated samples using a Qiagen Plus mini kit were fragmented into 250-300 bp, end-repaired and adenylated at the 3' end to allow for ligation of Illumina adapters. Library fragments were amplified by initial denaturation at 98°C for 10 s, followed by 8 cycles of 98°C for 10 s, 60°C for 30 s and 72°C for 30 s, and final extension step for 5 min at 72°C. During amplification, each sample was barcoded with a distinct adapter to allow multiplex sequencing. RNA libraries were quantitated by qPCR (KAPA Biosystems), pooled in equimolar quantities, and sequenced on an Illumina HiSeq2500 to generate paired-end reads.

ScRNA-seq was performed on NTERA-2 (ECSCs, DNLCs) cells by thawing approximately 1 million cryopreserved cells and resting in PBS, followed by centrifugation at 400 *xg* for 7 min. The supernatants were discarded, and cells were resuspended in PBS with BSA, and centrifuged again at 400 *xg* for 7 min. The dead ECSCs or DNLCs was immunomagnetically removed using the EasySep™ Dead Cell Removal (annexin V) kit. We loaded ~8,000 lives cells each from the negatively-selected suspensions onto the chip to recover ~5,000 cells. Each sample was diluted to a concentration of 700-1200 cells per µl and loaded into individual wells of the 10x Chromium chip following manufacturer's procedures. These includes generation of droplets with encapsulated cells, lysing each cell and reverse transcribing its RNA, and recovering the barcoded cDNA through bead purification and demulsification. The libraries from the pre-amplified cDNA generated using the manufacturer's instructions were sequenced to a depth of 50,000 reads per cell using a S4 flow cell run on the Illumina NovaSeq 6000 system.

Immunocytochemistry, Immunohistochemistry, and Confocal Imaging

Staining to examine the expression of stemness/neuronal markers was carried out as previously described (Malty et al., 2017). Coronal sections were blocked for 1 h in phosphate buffer (PB) solution (pH 7.4) containing 5% horse serum and 0.3% Triton X-100 (Bio-Rad). Proteins of interest were labeled with appropriate primary antibody at 4°C and incubated overnight. Slices were washed three times (10 min) and incubated for 1 h at room temperature with secondary antibody, followed by three 10 min washes in PB. After labeling the slices with DAPI and rinsing three times, it is mounted on gelatin-coated microscope slides and dried overnight at 4°C, followed by treating slices with ProLong Gold Antifade Tissue Mountant (Invitrogen) and sealing under glass coverslips. Imaging was performed using a Zeiss LSM700 laser scanning confocal microscope using a Plan-Apochromat 63X/1.4 oil objective lens. Z-stack images were taken at 1 µm intervals and data was collected using Zeiss Zen 2009 ver. 5.5 (Carl Zeiss).

IP and Immunoblotting

The lysates from NTera2 cells and mouse brain were adjusted to 1 ml with RIPA buffer (Malty et al., 2017) containing PIC and to which 3 µl of antibody was added. After 1 h agitation at 4°C, 100 µl of µMACS protein A magnetic microbeads (Miltenyi) was added with continued agitation for an additional 4 h at 4°C. Microbeads suspension was passed through µMACS columns (Miltenyi), washed 2 times with 1 ml of 0.1% RIPA and PIC, followed by another wash with 1ml detergent free RIPA. Proteins were eluted using 100 µl of 2x Laemmli buffer heated at 95°C. Eluates were analyzed by immunoblotting as described (Malty et al., 2017).

BN-PAGE and CI/III/IV Colorimetric Assays

About 200 µg of mt from control and *C20orf24* knockout (KO) DNLCs solubilized in a sample buffer (Thermo Fisher) with 2% digitonin was centrifuged at 20,000 *xg* for 10 min, followed by

separation of mt respiratory complex assemblies on NativePAGE™ 3-12% Bis-Tris gel at 150V for 30 min and 250V for 150 min. Gel was stained and destained using standard BN-PAGE protocol. The respirasome activity was measured either in DNLCs or in healthy or CIV-deficient fibroblasts (CDFs) transfected with constructs described in Figures 5 and S6, along with appropriate controls, using Mitocheck CI/III/IV kits following the manufacturer's protocol. By calculating the slope from the kinetic measurements of experimental samples over control, the fold change of respirasome activity was determined.

Oxygen Consumption Rate (OCR) and Extracellular Acidification Rate (ECAR)

DNLCs stably expressing *C20orf24* KOs or non-targeting control sgRNA were seeded in Agilent Seahorse XF96 V3 PS culture microplates at a density of 9,000 cells per well. After 24 h, the growth media was replaced with DMEM and processed in the Agilent Seahorse XFe96 analyzer to test for OCR and ECAR. For OCR measurement, cells were treated with a final concentration of 1 μM oligomycin, followed by 0.25 μM FCCP, as well as 0.5 μM rotenone and 0.5 μM antimycin A. In the case of ECAR, the growth media was replaced with Agilent Seahorse XF base medium, and the cells sequentially treated with 10 mM glucose, 1 μM oligomycin, and 50 μM 2-deoxyglucose were analyzed using the Agilent Seahorse Wave software.

Structural Modeling and Docking

Iterative Threading ASSEmbly Refinement (I-TASSER) was used to predict unresolved structures of C20orf24 and NENF proteins. Structure with lowest energy was refined using a fragment-guided molecular dynamic procedure to optimize hydrogen-bonding and remove steric clashes. The resolved human structures of DJ-1 (3SF8), CI (5XTD), CIII (5XTE), and CIV (5XTH) were obtained from protein data bank. The protein models were prepared using the Schrödinger modeling software, and the root mean square deviation was set to 0.3 Å with any

deviation below this was considered negligible. The docking analyses was carried out using PIPER, and for high-scoring docked structure, residues at the complex interface was determined using Schrödinger to measure the change in solvent-accessible surface area between bound and unbound forms of two proteins. Residues with a minimum 15% solvent-accessible surface area in the unbound state were considered as a potential interface between interacting proteins.

Computational Analysis of Off-target Effects of sgRNAs Targeting Select Genes of Interest

Potential off-target effects of sgRNAs designed to target the genes (see Table below) was computationally analyzed using Cas-OFFinder (Bae et al., 2014) and CHOPCHOP (Labun et al., 2019). We found that each of the chosen sgRNAs (results from Cas-OFFinder shown) has a single hit in the human genome with perfect complementarity to its target gene. With the exception of sgRNAs for PDHA2 or PINK1, all other sgRNAs had off-targets with 3 mismatches between sgRNA and genomic DNA, indicating low efficiency of editing. Conversely, CHOPCHOP showed no mismatches or off-target genes. These results suggest that chosen sgRNAs are highly selective and efficient. Nevertheless, to avoid the effects from off-target genes, wherever the gene of interest has been disrupted via CRISPR/Cas9, an add-back/rescue experiment was performed by overexpressing the CRISPR-resistant form of the gene.

Gene	CRISPR sgRNA	DNA targets (mismatches in small case)	Chromosome (Position/ Direction)	Mis matches	Gene at this locus	Seed region mismatch (Y/N)
C20orf24	TGGACCGCGAATCAGTGTGTNGG	TGGACCGCGAATCAGTGTGTTGG	chr20 (36612167/+)	0	C20orf24	N
		TGGACCGCGAATCAGaGccTCGG	chr5 (151566267/-)	3	FAT2	Y
		TGaACaGCcAATCAGTGTGTAGG	chr1 (93923371/+)	3	MTND4P11	N
		TGGAgCGgGAAaCAGTGTGTAGG	chr1 (170669618/-)	3	PRRX1	Y
		TGGACCcCcA				

		ATgAGTGTGTTGG	chr20 (53797878/+)	3	intergenic region	Y
DJ-1	AGTACAGTGTAGCCGTGATGNGG	AGTACAGTGTAGCCGTGATGTGG	chr1 (7965361/+)	0	DJ-1	N
		AGTtCAGTGTAaCCGTGgTGGGG	chr8 (137806036/+)	3	intergenic region	Y
		AGTACAGgGTAGatGTGATGAGG	chr1 (56311500/-)	3	RNA gene (AC119674.1)	Y
		AGTACAGTGaAGCCGTGgTtGGG	chr20 (58234162/-)	3	PPP4R1L	Y
		AGTACAGTGTAtaCGTGATtAGG	chr18 (74900133/+)	3	ZNF407	Y
		AGTgCAGTGTgGCCcTGATGTGG	chr11 (117203117/+)	3	TAGLN	Y
NENF	ACTACAGAGTTTTATGGACGNGG	ACTACAGAGTTTTATGGACGAGG	chr1 (212444331/+)	0	NENF	N
		ACTACAtAGgTTTATGGACaAGG	chr3 (24603810/-)	3	RNA gene (THRB-AS1)	Y
		ACcACAGAGTTTTATtGAaGGGG	chr1 (48784413/-)	3	AGBL4	Y
		ACTACAGtGTTTTcTGGACtGGG	chr11 (17958547/+)	3	SERGEF	Y
PINK1	CGCCACCATGGCGGTGCGACNGG	CGCCACCATGGCGGTGCGACAGG	chr1 (20633541/+)	0	PINK1	N
PDHA2	ATGTAATGACGTGATCCGAGNGG	ATGTAATGACGTGATCCGAGGGG	chr4 (95840470/-)	0	PDHA2	N

Generation of KOs and Mutagenesis

CRISPR KOs were generated using the oligonucleotide pairs encoding 20-nt guide RNAs (sgRNAs) targeting sequence for genes encoding interacting proteins using the standard procedure we described earlier (Malty et al., 2017). For selecting *PINK1* or *DJ-1* single KOs, puromycin-resistance selectable marker was used, whereas *NENF* KO was with blasticidin resistance. To generate *PDHA2* phosphomimetic and non-phosphorylatable mutants, sequence

verified plasmid encoding cDNA of PDHA2 (HsCD00347415) from the human ORFeome collection (Dana Farber/Harvard Cancer Center DNA Resource Core) was cloned using the forward and reverse PDHA2 primers into pLEX-307 at the NheI and SpeI restriction sites.

The canonical protospacer adjacent motif (PAM) GGG was mutated to GGA, rendering the PAM site resistant to Cas9 recognition. The pLEX-307-PDHA2 was cut using BsrGI and BmgBI, gel purified, annealed with PDHA2-CR (CRISPR-resistant) oligos, and ligated to generate CR PDHA2 (pLEX PDHA2-CR). This CR PDHA2 was cut using BstXI and BamHI, and the resulting fragments were annealed using the oligos of PDHA2-mut1 and 2, PDHA2-mut3 and 4, which was combined with either PDHA2-mut S291D/S293D (phosphomimetic), or PDHA2-mut S291A/S293A (non-phosphorylatable) listed in **Key Resource Table**. Cut vector and annealed oligos 1 and 2, 3 and 4 and each of the phosphomimetic or non-phosphorylatable mutants were annealed at 16°C overnight. Ligation products were transformed into bacteria and colonies selected. All mutants were verified by sequencing at TCAG.

Additionally, sequence verified Gateway cloning-compatible plasmid (pENTR223) encoding cDNA of DJ-1 (HsCD00383508) from the human ORFeome was mutated using the PCR-driven overlap extension method (Malty et al., 2017). Briefly, two sets of PCR reactions were carried out using DJ-1 outer forward and inner reverse primers corresponding to the desired mutation. For each mutation, a third PCR reaction was run using the forward and reverse PCR reactions mixed along with the outer primers. After digesting the PCR product or plasmid with BsmBI and XbaI, the purified amplicons and plasmid were ligated and sequence verified. Wild type or mutant *DJ-1* was then Gateway cloned into the plasmid pLD-puro-CcVA (Addgene), with a C-terminal versatile affinity (VA) tag containing 3× Flag, 6× histidine, and 2× Streptactin

epitopes (Flag and His separated by dual tobacco etch virus protease cleavage sites). After confirming the resulting clones by sequencing at TCAG they were transfected into *DJ-1* KOs.

Next, the 3’UTR C20orf24 variant was generated using the C20orf24-outer_F, C20orf24-outer_R, C20orf24-inner_F and C20orf24-inner_R primers, and followed the procedure as previously described (Heckman and Pease, 2007). To construct vectors encoding the C20orf24 coding sequence with the ectopic expression of wild type or (c.398G>G) variant (c.*398G>A) 3’UTR of C20orf24 in healthy fibroblasts, we used C20orf24-3'UTR-transf_F and C20orf24-3'UTR-transf_R primers to PCR amplify the wild type or variant 3’UTR of C20orf24. Plasmid (pPHAGE-CMV-C20orf24-C-TAP) encoding the C20orf24 coding sequence (Dana Farber, HsCD00448817) after digesting with EcoRI was combined with the above PCR fragments at appropriate molar ratios, ligated using Gibson assembly, and transformed colonies validated using Sanger sequencing. To generate PINK1-CR, silent synonymous mutations to the PAM site was introduced using PINK1-outer_F, PINK1-outer_R, PINK1-inner_F, and PINK1-inner_R primers, while NENF-CR was created with silent synonymous mutations to the sgRNA seed region in Phe81, Tyr82, Gly83 and Arg84 using a gBlock (IDT) designed with desired mutations. All the resulting mutants were verified by Sanger sequencing. C20orf24, PINK1, and NENF constructs obtained from Dana Farber Plasmid ID repository were c-TAP-tagged, which contains hemagglutinin A (HA) and FLAG epitope tags.

PDH (or PDHA2) Activity and Glutamate Excitotoxicity

The assay mixture contained 25 mM sodium phosphate (pH 7.8), 0.1% digitonin, 0.2 mM CoA, 0.5 mM pyruvate, 0.5 mM NAD, 0.2 mM thiamine pyrophosphate, 1 mM $MgCl_2$, 1 mM DTT, and 50 μL of mt extract from the ECSC or DNLC cultures, or cells stably expressing the wild type *PDHA2,* and phosphomimetic or non-phosphorylatable *PDHA2* mutants in *PDHA2* KO.

The enzyme reaction for PDH (or PDHA2) activity was carried out by incubating the assay mixture every 10 min up to 60 min at 37°C. After terminating the reaction at each time point by the addition of 50 μL of 25 % 5-sulfosalicylic acid, the assay mixture was centrifuged at 12,000 *xg* for 5 min and the supernatant (50 μl) was injected directly into the Agilent 1100 HPLC system. The method employed a Tosoh TSK-GEL ODS-100V column (250 × 4.6 mm i.d., particle size 5 μm) eluted with 100 mM NaH_2PO4 and 75 mM CH_3COONa (pH adjusted to 4.6 by the addition of H_3PO_4)-acetonitrile (94:6, v/v) at a flow rate of 1.0 ml/min, and an ultraviolet detector set at 259 nm. The standards of CoA and acetyl-CoA were eluted at roughly 11.0 and 25.1 min, respectively, and the enzymatic reaction product was quantified using a calibration curve built with acetyl-CoA. The specific activity of PDH (or PDHA2) was also measured following the method as previously described (Zhou et al., 2008). Susceptibility of ECSC and DNLC cultures expressing the aforesaid constructs to glutamate excitotoxicity was determined using the protocol described previously (Younkin et al., 1993).

Quantitative Real-time PCR

Total RNA was extracted either from the CDFs of two subjects (F-MT6119, F-MT2979) or transfected with constructs (described in Figures 5 and S6) in healthy or CDFs using the RNeasy Mini Kit (Qiagen) as per the manufacturer's protocol. The cDNA was synthesized from 50 ng of total RNA isolated from the aforesaid samples using the M-MLV reverse transcriptase and C20orf24 or beta actin (control) specific primers. PCR reaction was set up using the cDNA mixture, C20orf24 (or beta actin) primers, water, and LUNA universal SYBR green mix. The cycling parameters includes initial holding at 95°C for 10 min, followed by 60 cycles at 95°C for 25 s and 60°C for 1 min. The expression level of each construct normalized to the housekeeping gene was expressed as mean $2^{-\Delta CT}$ or $2^{-[CT\ (construct)\ -\ CT\ (ACTIN)]}$.

siRNA or miRNA Transfection

Healthy or CDFs transfected with pHAGE-eGFP (Harvard Cancer Center DNA Resource Core), C20orf24-FLAG overexpression plasmid (HsCD00448817) from the human ORFeome collection, and with constructs described in Figures 5 and S6, along with controls, was performed using lipofectamine RNAiMAX following manufacturer's instructions. Cells were harvested after 48 h incubation for total RNA and mt extraction.

Quantification of Cellular Heme and NENF Secretion

About 100 µg of proteins from the extracts of DNLCs was used to measure heme production in Synergy plate reader at an absorbance of 400 nm using Sigma's heme assay kit. The NENF secretion from the supernatant of cultured ECSCs or DNLCs (5×10^6 cells) in opti-MEM1 reduced-serum medium with 2% FBS was measured in Synergy plate reader at an absorbance of 450 nm using the human NENF enzyme-linked immunosorbent assay kit (G-Biosciences). The amount of NENF secreted was calculated using a four-parameter logistic curve fit.

Neurotrophic and Apoptotic Assays

Cell viability was assessed following the tetrazolium MTT (3-(4, 5-dimethylthiazolyl-2)-2, 5-diphenyltetrazolium bromide) cell proliferation kit (ATCC) by plating NTera2 DNLCs (5×10^3 cells) and measuring the absorbance (formazan) at 570 nm using a Synergy microplate reader. Neurite outgrowth was examined by plating DNLCs onto 6-well plates coated with poly-L-lysine (20 µg/ml) at a density of 1×10^4 cells/well in DMEM supplemented with 2% FBS. DNLCs were stained with mitotracker, exposed to β3-tubulin primary antibody and counterstained with Alexa 488 anti-mouse secondary antibody. The cells were imaged using the Zeiss Observer Z1 inverted microscope with Colibri 2 epifluorescence, and analyzed for the extension of dendrites and axons with at least two cell body lengths in diameter to score for a positive neurite outgrowth.

The caspase-9 activity was measured using luminescent caspase-Glo 9 assay following the manufacturer's procedure (Promega), periodically for 3 h using Synergy luminescence plate reader. Likewise, the BCL-2 activity was determined by treating DNLCs, plated at a density of 1 x 10^4 cells, with BCL-2 primary antibody for 1 h followed by incubation with Alexa 488 anti-rabbit secondary antibody for 1 h, and with Hoechst 33342 for 10 min. The cells were then read using synergy plate reader at 360 nm for Hoechst and 480 nm for BCL-2 expression level.

QUANTIFICATION AND STATISTICAL ANALYSIS

Data Filtering, Scoring mtPPIs, and Defining MPCs

Prior to generating the high-quality interactions using the integrated log-likelihood scores (ΣLLS), the MS/MS spectra of each biochemical fraction collected from two distinct cell states (ECSCs, DNLCs) was searched against the reference human target-decoy protein sequences (UniProt). As we previously described (Wan et al., 2015), to improve peptide-spectral matching sensitivity and accuracy, we used Sequest (ver. 27 - rev.9) and MS-blender (encompassing X! Tandem ver. 2013.09.01, Comet ver. 2016.01 rev. 3, MS-GF+ ver. 2017.01.13) search engines with default parameter settings to define high-confidence peptide-spectral matches at <1% FDR. Search parameters were set to allow for two missed cleavage sites, precursor mass tolerance ranged from -2 to 4 Da, variable modification of methionine oxidation, protein N-terminal acetylation, and one fixed modification of cysteine carbamidomethylation.

The Sequest search results were evaluated by the Statquest probability algorithm, whereas matches from the other three search engines were integrated with MS-blender, which uses a probabilistic approach to extract the best hits detected by each search method for integration. Prior to scoring, we retained only proteins from both replicates of the same elution techniques (SEC, IEC), and filtered proteins present in only one fraction or more than half of the eluted

fractions. The co-fractionation profile from each replicate of SEC or IEC was scored using PCC, WCC, and co-apex metric as previously reported (Havugimana et al., 2012; Wan et al., 2015). By compiling the curated human protein complexes containing mtPPIs from CORUM database as a reference set, true positive (interacting proteins annotated within the same complex) and true negative PPIs (interacting proteins annotated between complexes) were defined.

For all protein combination pairs, we considered only a PCC or WCC score of at least 0.5 or co-apex of 1, as we reported previously (Havugimana et al., 2012; Wan et al., 2015) to compute the LLS (Babu et al., 2018) against the reference dataset using the following formula:

$$LLS = ln\frac{P(L|E)/{\sim}P(L|E)}{P(L)/{\sim}P(L)}$$

Where *P(L|E)* denotes the frequency of interactions (L) in dataset E (i.e. from each replicate of SEC-MS or IEC-MS) containing the true-positive set, while *~P(L|E)* signifies the frequency of interactions L in the true-negative set. *P(L)/~P(L)* represents the prior odds ratio of true-positive and true-negative PPIs in the reference set. The LLS was computed within a cell state independently for each pairwise interaction in each replicate of SEC-MS or IEC-MS, and then used a weighted sum to produce a final score by averaging all replicates across techniques. The resulting 145,797 scored interactions in DNLCs, and 110,478 in ECSCs that was benchmarked using ROC analysis allowed us to choose a ΣLLS cut-off of ≥1.45 to generate 3,567 high-confidence mtPPIs in DNLCs, and 3,320 in ECSCs, respectively.

To validate interactions in ECSC or DNLC network (**Table S2**), we performed IP/MS (Malty et al., 2017) experiments using antibodies targeting 37 endogenous proteins in the mt extracts of mouse brain lysates, 21 from NTera2 DNLCs, and 79 from SH-SY5Y neuronal cells. Additional validation includes PPIs from BF/MS (84 fractions from SEC; 96 from IEC) performed in duplicate with 200 µg of mt extracts isolated from the mouse brain. As well,

alternate SEC method was conducted by the group of Leonard Foster (British Columbia, Canada) using a reduced quantity of mt extracts (100 µg) from DNLCs and fractionated using 300 x 7.8 mm BioSep4000 Column (Phenomenex) equilibrated with different SEC mobile phase (50 mM KCl, 50 mM $NaCH_3COO$, pH 7.2) than described above. The samples were separated into 81 fractions in triplicate, for a total of 243 fractionations by an Agilent 1200 series semi-preparative HPLC at a flow rate of 0.5 ml/min at 8°C. The trypsin digested fractions were analyzed using an Orbitrap Elite mass spectrometer, and the resulting MS spectra was acquired and processed as recently described (Malty et al., 2017). Lastly, using mtPPIs from DNLC network, we predicted putative MPCs by CoreMethod algorithm (Leung et al., 2009) with at least one MP, which was overlapped with mtPPIs from ECSC to identify the extent of rewiring within a complex.

Bulk RNA-seq and ScRNA-seq

The raw sequence reads (in FASTQ files) of the DNLCs or fibroblasts of CDFs and healthy individuals were processed using the Kallisto software. The resulting count value X_i for transcript *i* was normalized to CPM_i (Counts per Million) using the formula: $\frac{X_i}{N} \times 10^6$; where N is the number of sequenced fragments. The fold change between DNSCs and ECSCs, as well as between CDFs and healthy samples, and their corresponding Benjamini-Hochberg (BH) adjusted *p*-value for each differentially expressed transcript was computed using DESeq2 in R package.

Cell Ranger from the Chromium Single Cell Software Suite (ver 3.0.2; 10x Genomics) was used to demultiplex the raw base call files from the sequencer; extract, filter, and correct cell barcodes and unique molecular identifiers (UMIs); and remove cDNA PCR duplicates. Cell Ranger was then used to align the single cell sequencing reads (*.fastq.gz) generated for ECSCs and DNLCs to the human reference transcriptome (Sequence: GRCh38). The raw UMI (unique molecular identifiers) counts from each cell state were then aggregated using the ‘cellranger

aggr' pipeline into one unified gene-cell matrix file. The aggregation process avoided the batch effects by normalizing the read depth between cell states. The Bioconductor 'scran' (ver 1.12.0) package was used to filter empty droplets, low quality cells (i.e. large proportion of mitochondrial UMI counts above the median of all captured cells), cell multiplets, and genes not expressed in any cells, resulting in a final dataset consisted of 6,583 single cells (3,631 ECSCs, 2,952 DNLCs). UMI counts for each transcript in each cell was measured, and the total number of UMI counts were then averaged from all cells for a given transcript in each cell state. Differential gene expression analysis was calculated between DNLCs and ECSCs datasets, and BH adjusted p-value (i.e. $q \leq 0.01$) was considered significant.

The cell subtype assignments for 2,952 DNLCs was conducted by implementing a shared nearest neighbour-cliq (Xu and Su, 2015), followed by graph-based community detection algorithm (i.e. Walktrap) to identify putative clusters. We then performed pairwise differential gene expression analysis using Welch's t-test for each cluster relative to all other clusters, and chose top 50 significantly ($P \leq 0.05$) ranked genes (with $\log_2$ fold change greater than 0) as gene markers for a given cluster. These were then mapped to known neuronal or glial marker genes from the recently published studies (Lake et al., 2016; Zhong et al., 2018) to assign distinct cell subtypes. Each differentially expressed genes from DNLCs to a major cell subtype was assigned only when their expression level is significantly ($q \leq 0.05$) higher than in other cell subtypes.

Identification of High-Confidence Phosphosites

Peptides were identified using Sequest and filtered at a stringent 1% FDR using the target-decoy approach based on XCorr (>1.5 for +2, +3 and +4 charged precursor ions) and mass accuracy (< 10 ppm) parameters. Each phosphosite was scored using Ascore (Beausoleil et al., 2006), which measures correct phosphosite based on the intensity of site-determining ions in MS/MS spectra.

Only phosphosites with Ascore ≥13 ($p \leq 0.05$) and measured in at least two of the three replicates were considered as genuine. In few cases where phosphosites measured in one replicate, exception was made as long as they are detected in PhosphoSitePlus database.

Enrichment Analyses

To identify which bioprocesses the proteins interacting with orphans were enriched in static or DF networks, we used g:Profiler that executes a gene-set enrichment analysis to identify statically enriched annotations on a seed gene list using hypergeometric significance estimation. We considered a process to be enriched when the adjusted *p*-value was less than 0.05 (or $q \leq 0.05$) after BH correction. Disease enrichment among the high-confidence phosphoproteins during differentiation was performed by downloading disease annotations from HGMD (Human Gene Mutation Database), genetic variants (single nucleotide polymorphism; SNP) from Uniprot, and disease entries from OMIM ID to disease ontology database. After integrating SNPs and HGMD, we annotated each phosphosite with all non-synonymous mutations that had at least 7 amino acid positions away from the modification site. Disease terms, computed using a Fisher's exact test after BH correction ($q \leq 0.05$) were considered significant.

Kinase-Substrate Network

Kinase-substrate relationships during differentiation were identified by combining all known kinase-substrate interactions from the PhosphoSitePlus database and predicting additional kinase-specific phosphorylation site by GPS 3.0 software with the threshold parameter set to high (FDR 2% for serine/threonine kinases, FDR 4% for tyrosine kinase). We allowed only a kinase-substrate interaction that was either specific or common to each cell state.

Statistical Tests

The results from the functional assays were obtained with enough statistical power by including a minimum of three independent biological and/or technical replicates to draw reasonable conclusions. Significance was determined using standard statistical tests, and wherever possible, non-parametric tests, mean ± SD and number of replicates were outlined in the figure legends.

DATA AND SOFTWARE AVAILABILITY

All raw proteomic data from this work is submitted to the PRIDE repository (Accession: PXD009831-34) at the European Bioinformatics Institute, in accordance with the data sharing policy. As well, scRNA-seq and bulk RNA-seq datasets were deposited at NCBI sequence read archive repository (PRJNA474206). Codes used in generating the results are aforesaid in detail.

SUPPLEMENTARY REFERENCES

Babu, M., Bundalovic-Torma, C., Calmettes, C., Phanse, S., Zhang, Q., Jiang, Y., Minic, Z., Kim, S., Mehla, J., Gagarinova, A., *et al.* (2018). Global landscape of cell envelope protein complexes in Escherichia coli. Nat. Biotechnol. *36*, 103-112.

Bae, S., Park, J., and Kim, J.S. (2014). Cas-OFFinder: a fast and versatile algorithm that searches for potential off-target sites of Cas9 RNA-guided endonucleases. Bioinformatics *30*, 1473-1475.

Beausoleil, S.A., Villen, J., Gerber, S.A., Rush, J., and Gygi, S.P. (2006). A probability-based approach for high-throughput protein phosphorylation analysis and site localization. Nat. Biotechnol. *24*, 1285-1292.

Havugimana, P.C., Hart, G.T., Nepusz, T., Yang, H., Turinsky, A.L., Li, Z., Wang, P.I., Boutz, D.R., Fong, V., Phanse, S., *et al.* (2012). A census of human soluble protein complexes. Cell *150*, 1068-1081.

Heckman, K.L., and Pease, L.R. (2007). Gene splicing and mutagenesis by PCR-driven overlap extension. Nat. Protoc. *2*, 924-932.

Kohda, M., Tokuzawa, Y., Kishita, Y., Nyuzuki, H., Moriyama, Y., Mizuno, Y., Hirata, T., Yatsuka, Y., Yamashita-Sugahara, Y., Nakachi, Y., *et al.* (2016). A Comprehensive Genomic Analysis Reveals the Genetic Landscape of Mitochondrial Respiratory Chain Complex Deficiencies. PLoS Genet. *12*, e1005679.

Labun, K., Montague, T.G., Krause, M., Torres Cleuren, Y.N., Tjeldnes, H., and Valen, E. (2019). CHOPCHOP v3: expanding the CRISPR web toolbox beyond genome editing. Nucleic Acids Res. pii: gkz365.

Lake, B.B., Ai, R., Kaeser, G.E., Salathia, N.S., Yung, Y.C., Liu, R., Wildberg, A., Gao, D., Fung, H.L., Chen, S., *et al.* (2016). Neuronal subtypes and diversity revealed by single-nucleus RNA sequencing of the human brain. Science *352*, 1586-1590.

Leung, H.C., Xiang, Q., Yiu, S.M., and Chin, F.Y. (2009). Predicting protein complexes from PPI data: a core-attachment approach. J. Comput. Biol. *16*, 133-144.

Malty, R.H., Aoki, H., Kumar, A., Phanse, S., Amin, S., Zhang, Q., Minic, Z., Goebels, F., Musso, G., Wu, Z., *et al.* (2017). A Map of Human Mitochondrial Protein Interactions Linked to Neurodegeneration Reveals New Mechanisms of Redox Homeostasis and NF-kappaB Signaling. Cell Syst. *5*, 1-14.

Perciavalle, R.M., Stewart, D.P., Koss, B., Lynch, J., Milasta, S., Bathina, M., Temirov, J., Cleland, M.M., Pelletier, S., Schuetz, J.D., *et al.* (2012). Anti-apoptotic MCL-1 localizes to the mitochondrial matrix and couples mitochondrial fusion to respiration. Nat. Cell. Biol. *14*, 575-583.

Wan, C., Borgeson, B., Phanse, S., Tu, F., Drew, K., Clark, G., Xiong, X., Kagan, O., Kwan, J., Bezginov, A., *et al.* (2015). Panorama of ancient metazoan macromolecular complexes. Nature *525*, 339-344.

Wieckowski, M.R., Giorgi, C., Lebiedzinska, M., Duszynski, J., and Pinton, P. (2009). Isolation of mitochondria-associated membranes and mitochondria from animal tissues and cells. Nat. Protoc. *4*, 1582-1590.

Williamson, C.D., Wong, D.S., Bozidis, P., Zhang, A., and Colberg-Poley, A.M. (2015). Isolation of Endoplasmic Reticulum, Mitochondria, and Mitochondria-Associated Membrane and Detergent Resistant Membrane Fractions from Transfected Cells and from Human Cytomegalovirus-Infected Primary Fibroblasts. Curr. Protoc. Cell Biol. *68*, 3 27 21-33.

Xu, C., and Su, Z. (2015). Identification of cell types from single-cell transcriptomes using a novel clustering method. Bioinformatics *31*, 1974-1980.

Younkin, D.P., Tang, C.M., Hardy, M., Reddy, U.R., Shi, Q.Y., Pleasure, S.J., Lee, V.M., and Pleasure, D. (1993). Inducible expression of neuronal glutamate receptor channels in the NT2 human cell line. Proc. Natl. Acad. Sci. U S A *90*, 2174-2178.

Zhong, S., Zhang, S., Fan, X., Wu, Q., Yan, L., Dong, J., Zhang, H., Li, L., Sun, L., Pan, N., *et al.* (2018). A single-cell RNA-seq survey of the developmental landscape of the human prefrontal cortex. Nature *555*, 524-528.

Zhou, Q., Lam, P.Y., Han, D., and Cadenas, E. (2008). c-Jun N-terminal kinase regulates mitochondrial bioenergetics by modulating pyruvate dehydrogenase activity in primary cortical neurons. J. Neurochem. *104*, 325-335.

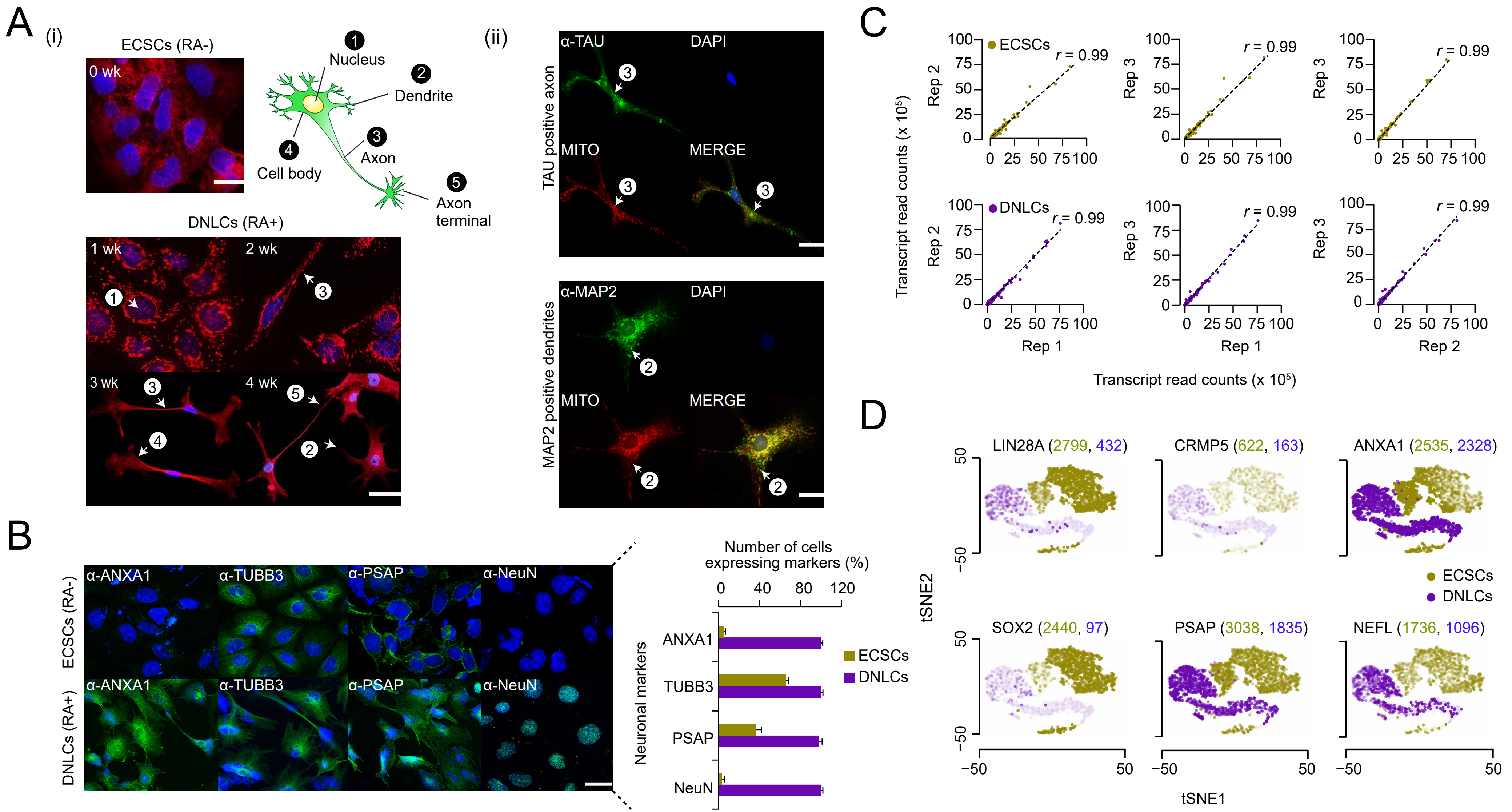

Figure S1. Morphological Characterization and Transcriptome Profile Changes in ECSCs and DNLCs. Related to Figure 1 and Transparent Methods.

(A) Micrographs portraying ECSCs in the absence (-) of retinoic acid (RA), and DNLCs with its various neuronal parts as indicated in the presence (+) of RA each week over a differentiation period of 4 weeks (i). Cells were stained with mitotracker (red) and DNA visualized by DAPI (blue). Images of DNLCs positive for Tau axonal and MAP2 dendritic markers (ii); scale bar, 10 μm.

(B) Micrographs and quantitation on the number of ECSCs and DNLCs (n = 300 cells per replicate; 4 weeks after differentiation) expressing neuronal markers. DNA visualized by DAPI (blue); scale bar is 20 μm. Data are mean ± SD (n = 3 technical replicates).

(C) Average correlation of all transcript read counts between replicates in each cell state.

(D) T-SNE plots showing the distribution of cell states (number of single cells shown in parentheses) for each of the indicated stemness/neuronal markers.

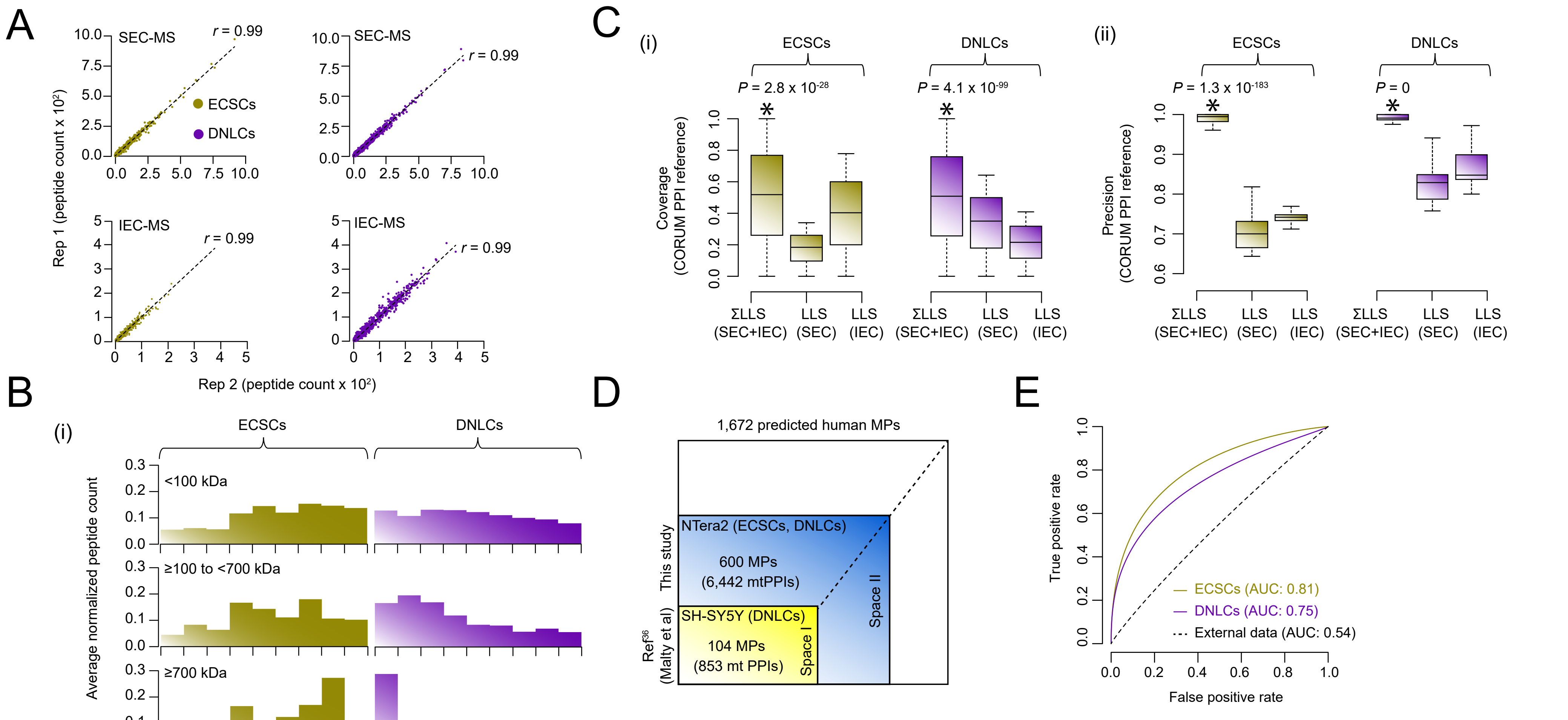

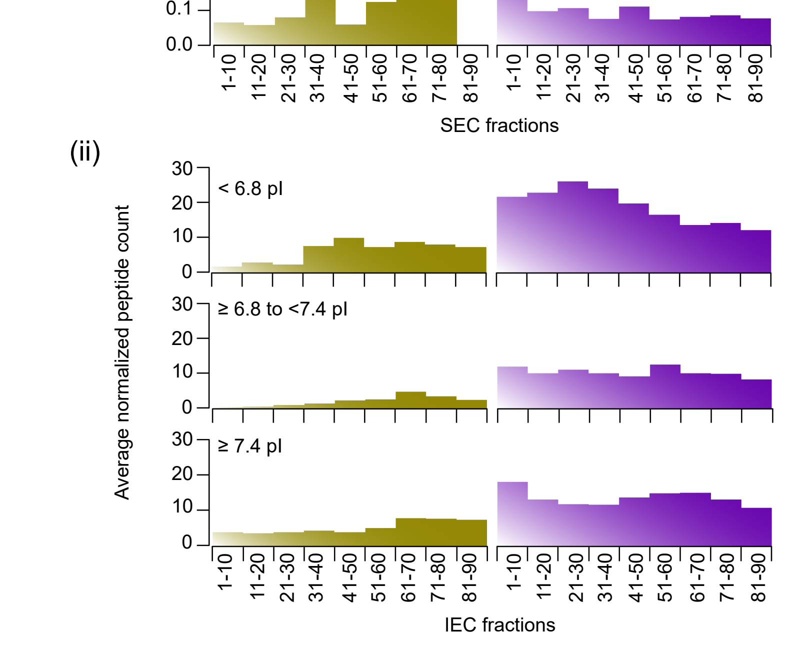

Figure S2. Co-elution profiles, scoring, and validating mtPPIs in ECSCs and DNLCs. Related to Figures 1-3 and Transparent Methods.

(A) Average correlation (peptide counts; ii) of replicate SEC- or IEC-MS analyses.

(B) Agreement of co-eluting proteins with molecular weights (kDa) or isoelectric points (pI) inferred from SEC (i) and IEC (ii) profiles.

(C) Coverage and accuracy of mtPPIs against reference CORUM complexes containing mt interacting proteins; **p*-value (ΣLLS vs LLS scores from SEC or IEC) computed by Wilcoxon signed-rank test.

(D) Experimental scale-up (blue; space II) relative to our previous (yellow; space I) human mt proteins and interactions derived from SH-SY5Y neuronal cells (Malty et al., Cell Systems, 2017).

(E) Peformance measures of the true positive rate vs false positive rate using 5-fold cross validation is shown by comparing area-under-the ROC (AUC) for co-complex interactions detected in ECSC or DNLC networks against external data derived from large-scale studies (references cited in Fig 2B).

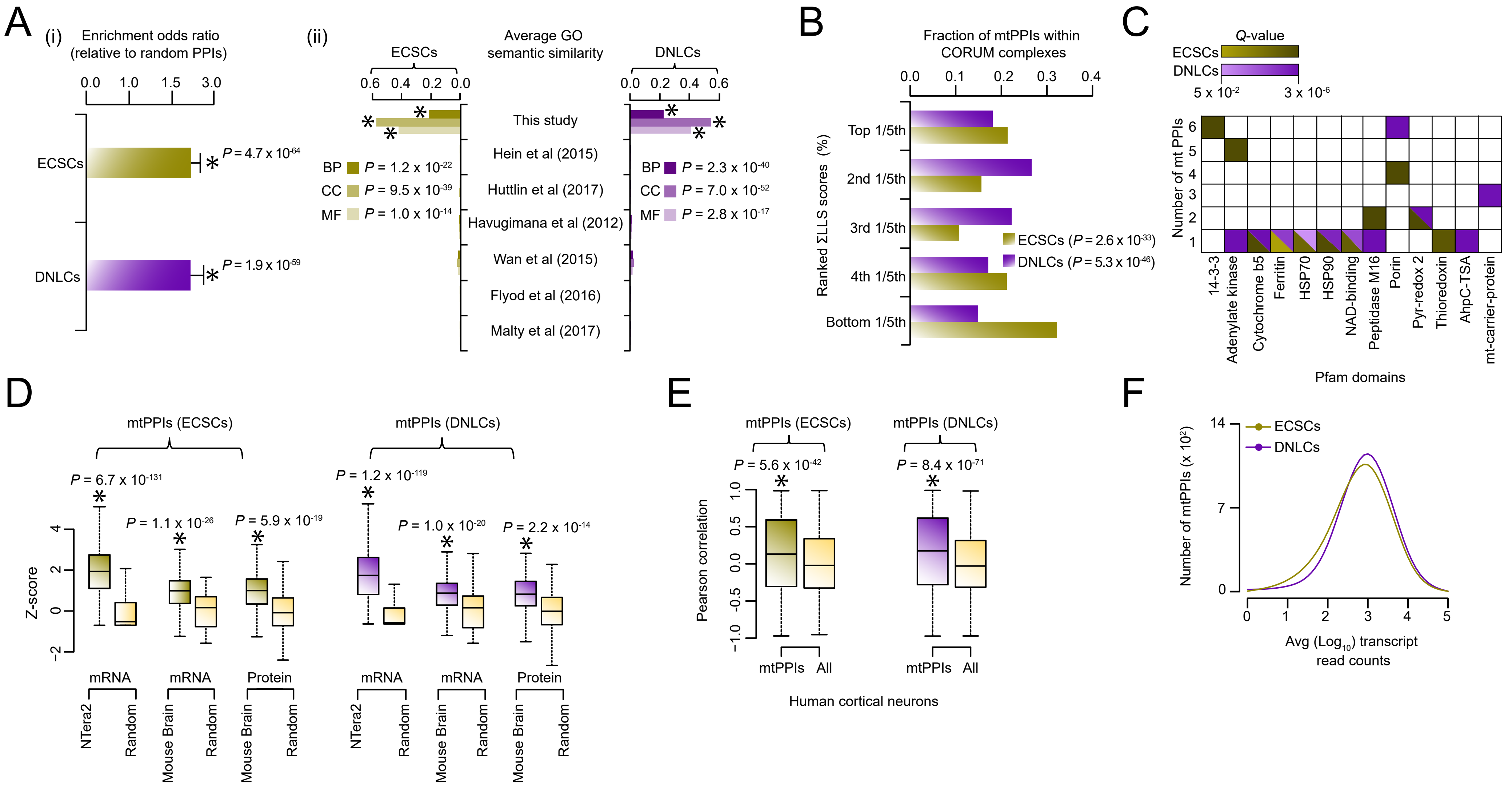

Figure S3. Benchmarking mtPPIs in ECSC or DNLC Network. Related to Figures 2-3 and Transparent Methods.

(A) Putative interacting proteins sharing phenotypic annotations (i) derived from MGI database (http://www.informatics.jax.org) of mouse orthologs compared to random set of protein interacting pairs, as well as to gene ontology (GO; ii) annotations (associated with BP, biological processes; CP, cellular components; and MF; molecular functions) compared to indicated large-scale PPI studies in ECSC or DNLC network. **p*-value by Fisher's exact test (i) and Student's *t*-test (ii).

(B) MtPPIs in top or bottom 20th percentile of ranked ΣLLS scores from ECSC or DNLC network enriched within the CORUM protein complex; **p*-values (top or bottom 20th percentile mtPPIs of ranked ΣLLS scores within CORUM complexes compared to randomly drawn PPI pairs) by Wilcoxon signed-rank test.

(C) Interacting proteins in ECSC or DNLC network enriched for shared Pfam domains (representative ones shown); False discovery rate (FDR; Benjamini-Hochberg correction) adjusted *p*-value (or *Q*-value) of the hypergeometric test.

(D) Box plots showing mRNA transcript and protein levels (Z-score transformed) of mtPPIs in ECSC or DNLC network vs randomly generated protein pairs (bootstrapped 1000 times) using RNA-seq (NCBI GEO: GSE52564; PMID: 25186741) and label-free quantification (PMID: 26523646) of mouse brain; **p*-values by Wilcoxon signed-rank test.

(E) Correlation of mt interacting proteins in ECSCs or DNLC network vs all protein pairs co-expressed in human cortical neurons (NCBI GEO: GSE56796; PMID: 24991954); **p*-values by Wilcoxon signed-rank test.

(F) MtPPIs in ECSC or DNLC networks is plotted against the mRNA transcript abundance derived from RNA-sequencing of Ntera2 ECSCs and DNLCs.

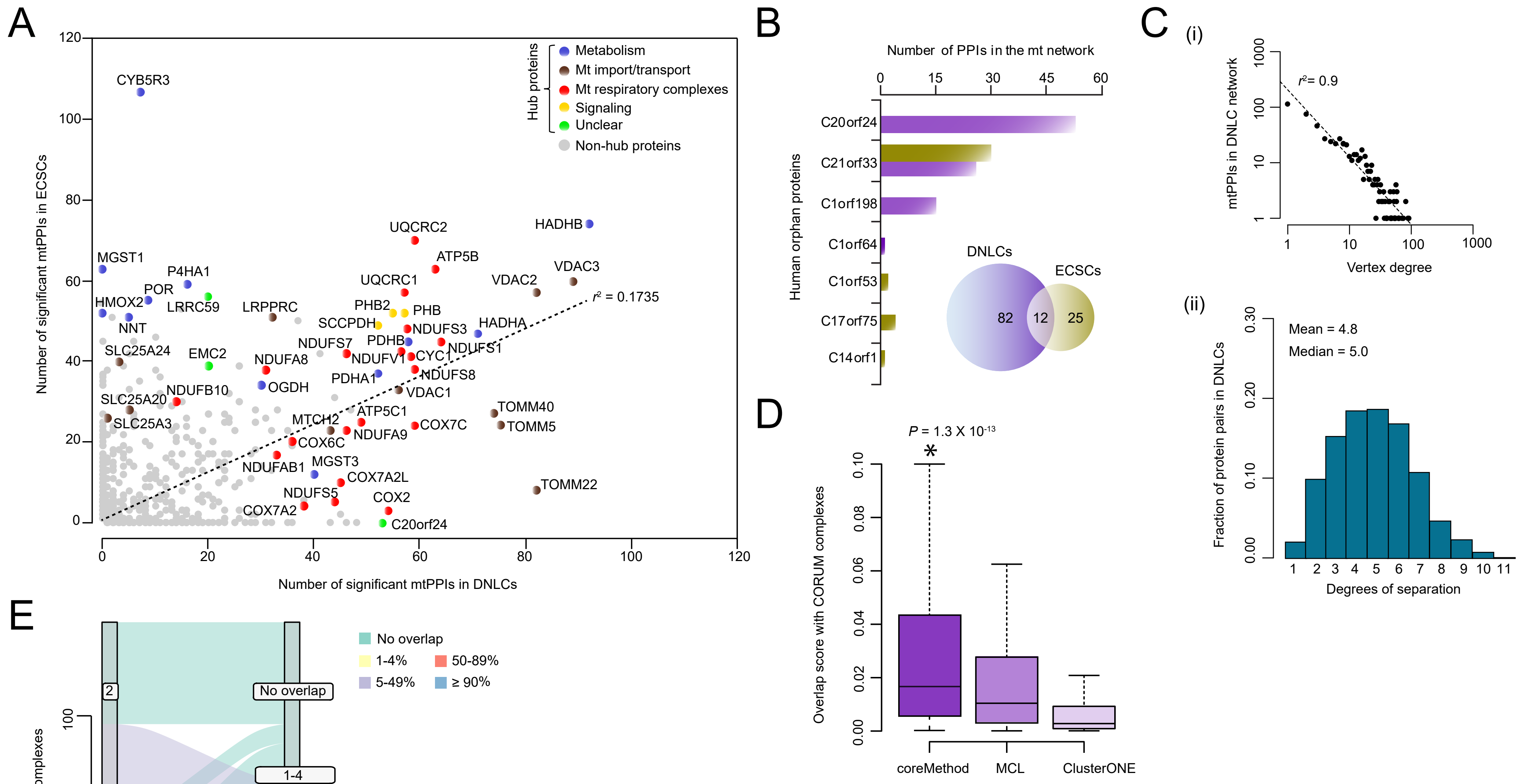

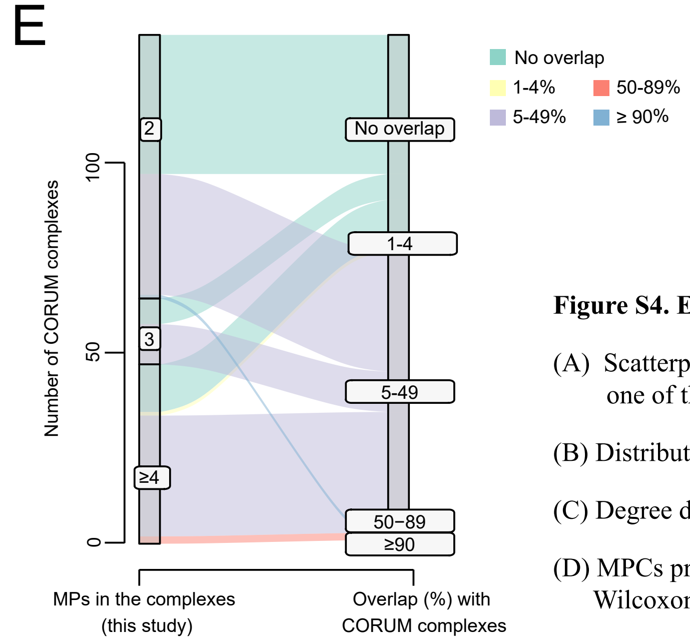

Figure S4. Evaluation of MPCs Predicted in DNLC Network. Related to Figures 2 and 3 and Transparent Methods.

(A) Scatterplot of representative hub proteins from the indicated process is shown based on connectivity with 50 or more MPs in either one of the static networks.

(B) Distribution of PPIs associated with orphan proteins and their overlap in static networks.

(C) Degree distribution (i) and seperation (ii) of interacting protein pairs in DNLC network.

(D) MPCs predicted by various clustering algorithms as indicated was compared against reference CORUM complexes; **p*-value by Wilcoxon signed-rank test.

(E) Maximum overlap between predicted MPCs and reference set of CORUM complexes.

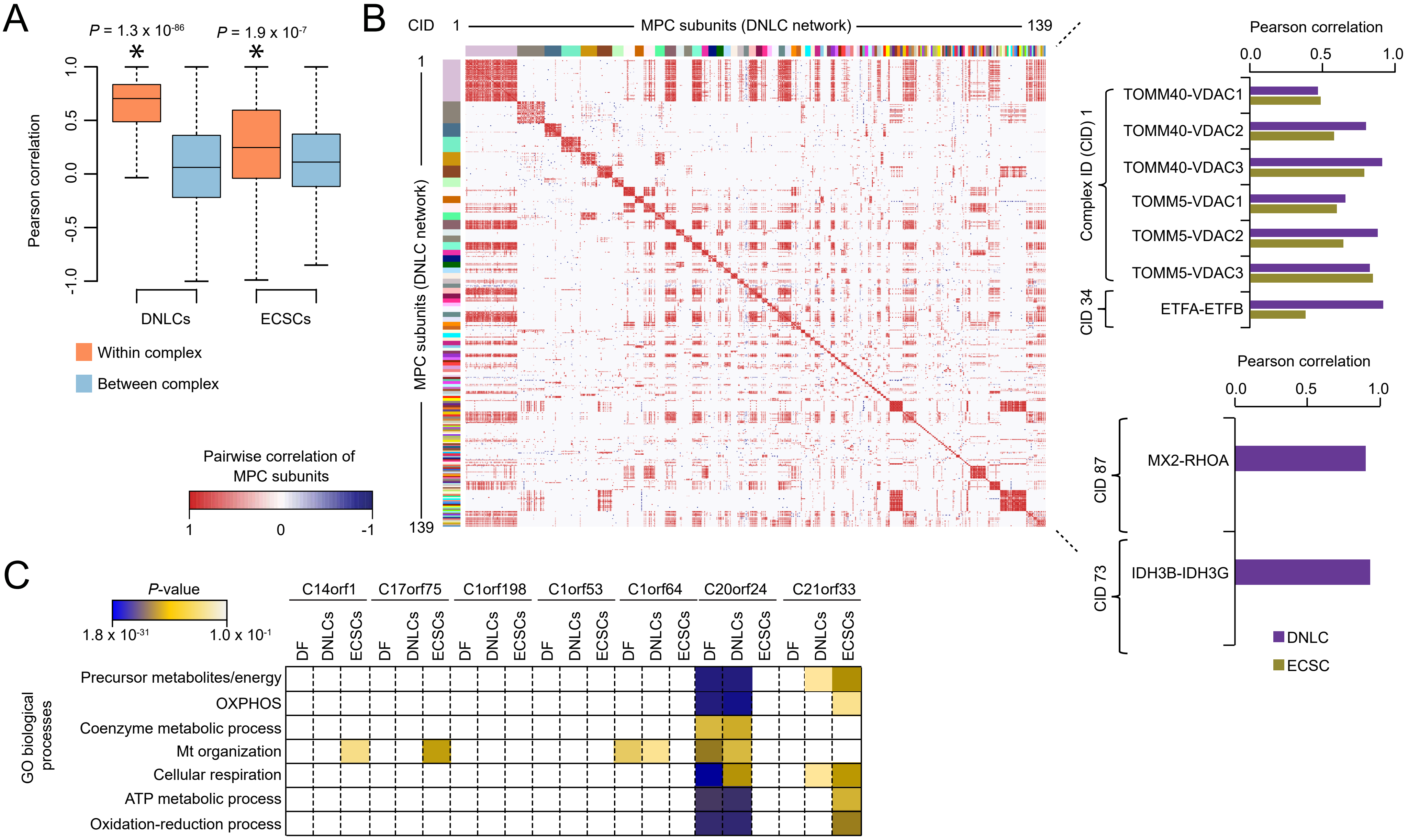

Figure S5. Evaluation of MPCs Predicted in Static and/or DF (Differential) Networks. Related to Figures 2 and 3 and Transparent Methods.

(A) Correlation of interaction profiles for proteins within and between complexes in ECSC and DNLC network; **p*-value by Wilcoxon signed-rank test.

(B) Symmetrical heat map showing pairwise correlation of interacting proteins using their respective LLS score within the MPC predicted in DNLC network (left). Bar graphs (right) show correlated profiles for protein interacting pairs within the indicated cluster.

(C) Orphan association with proteins from ECSC, DNSC, and DF networks enriched in representative Gene ontology (GO) biological processes as indicated; *p*-value by hypergeometric test.

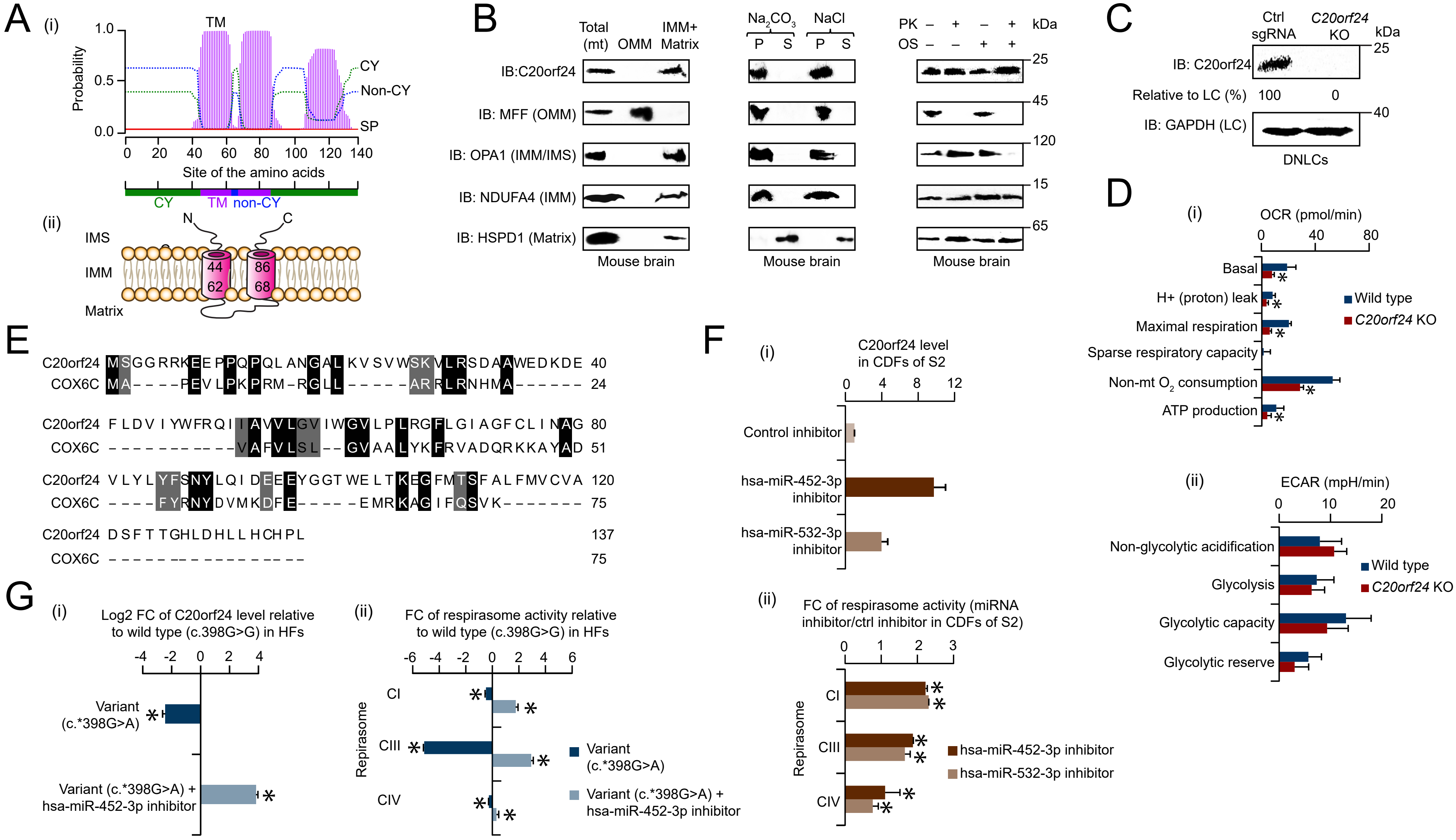

Figure S6. Characterization of C20orf24 and its Role in Respirasome. Related to Figure 5 and Transparent Methods.

(A) Transmembrane (TM) prediction for C20orf24 using Phobius (i) is shown with start and stop amino acid position of two transmembrane helix (ii) in the inner mt membrane (IMM); IMS, inter membrane space; SP, signal peptide; CY, cytoplasmic region.

(B) C20orf24 (along with mt compartmental markers as controls) in the indicated subcellular fractions of mouse brain, and in soluble (S) or membrane pellet (P) fraction of mouse brain extracts with NaCl or Na_2CO_3, as well as in the extracts of mouse brain treated with proteinase K (PK) in the presence of osmotic shock (OS) was immunoblotted (IB) using the indicated protein-specific antibodies; OMM, outer mt membrane.

(C) C20orf24 level in the control (ctrl) sgRNA and *C20orf24* CRISPR knockout (KO) of NTera2 DNLCs immunoblotted with anti-C20orf24 antibody. Band intensities normalized to GAPDH loading control (LC).

(D) Oxygen consumption rate (OCR, i) and extracellular acidification rate (ECAR, ii) measurements of KO and wild type cells; data represented as mean ± SD (n = 5 technical replicates; *$p \leq 0.05$ by Student's *t*-test).

(E) Amino acid sequence alignment of C20orf24 and COX6C using ClustalW software. Conserved sequences are shaded in black, while amino acid similarity groups (ILV, FWY, KRH, DE, GAS, TNQM) in grey.

(F, G) C20orf24 mRNA level and respirasome (Fold change, FC) activity measured by qRT-PCR or colorimetric assays in the CIV-deficient fibroblasts (CDFs) of subject 2 (S2, panel F) transfected with hsa-miR-452 or 532 miRIDIAN hairpin inhibitor vs. non-targeting miRIDIAN miRNA hairpin inhibitor control, or in healthy fibroblasts (HFs; panel G) transfected with variant (c.*398G>A) vs. wild type (c.398G>G), or variant (c.*398G>A) + hsa-miR-452-3p inhibitor vs. wild type (c.398G>G) or variant + non-targeting miRIDIAN miRNA hairpin inhibitor control (data shown for wild type c.398G>G); data represented as mean ± SD (n = 3-6 technical; *$p \leq 0.05$ by Student's *t*-test).

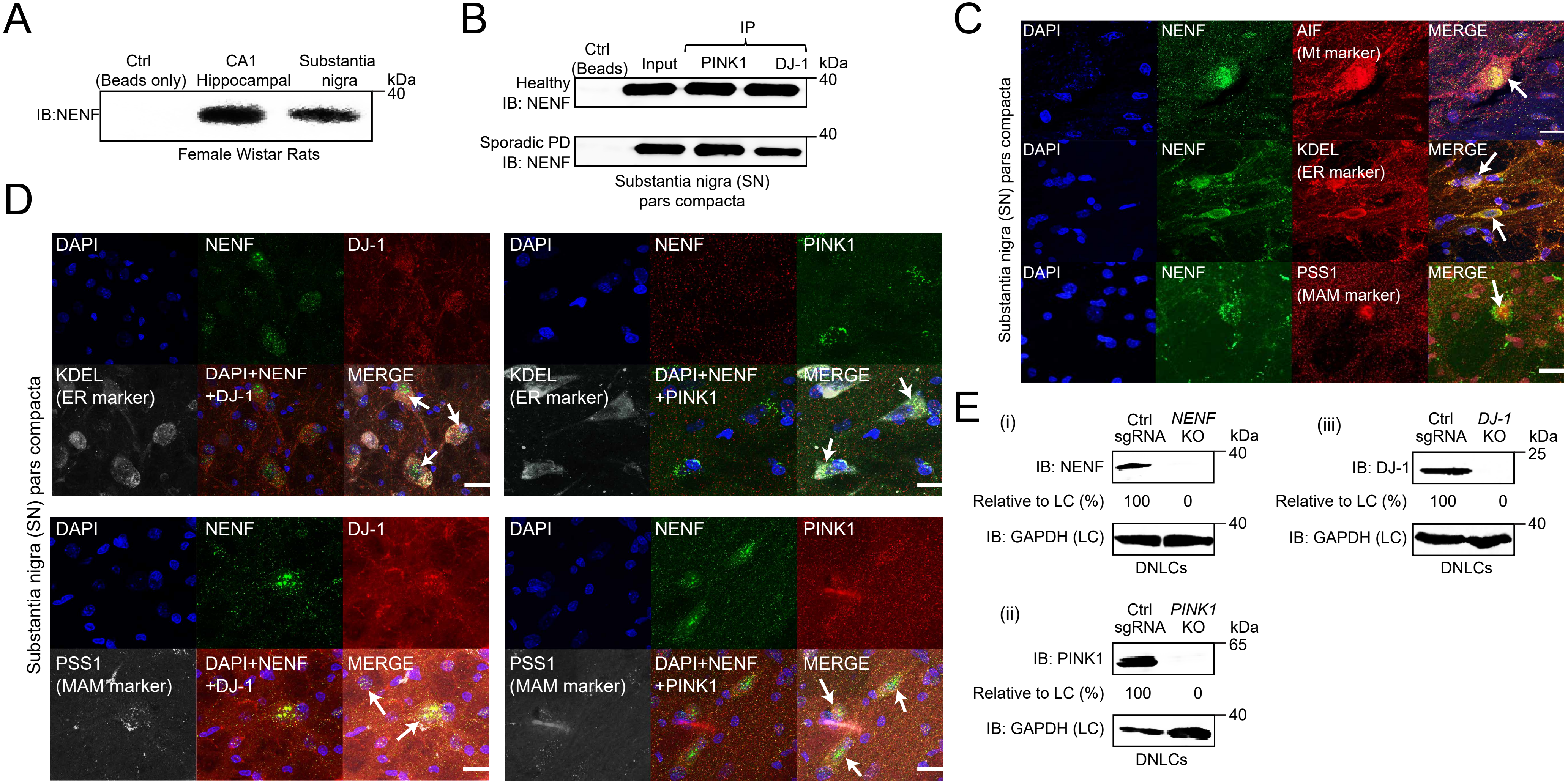

Figure S7. Characterization of NENF and its Association with DJ-1 and PINK1. Related to Figure 6 and Transparent Methods.

(A) Endogenous NENF level in the indicated brain regions of female Wistar rats; protein G beads alone (without antibody) served as negative control. Molecular masses (kDa) of marker proteins are indicated.

(B) PINK1 and DJ-1 immunoprecipitates (IPs) in the SN of healthy and sporadic PD patient using anti-PINK1 and DJ-1 antibodies were immunoblotted (IB) with anti-NENF antibody.

(C) Representative confocal micrographs of NENF localization (arrow heads) in the SN of rat brain using antibodies specific to NENF and indicated mt, ER, and MAM markers; DNA visualized by DAPI (blue), scale bar is 20 µm.

(D) Representative confocal micrographs of NENF colocalization (arrow heads) with DJ-1 and PINK1 in the SN of rat brain using antibodies specific to the indicated native proteins and ER or MAM markers; DNA visualized by DAPI (blue), scale bar is 20 µm.

(E) NENF (i), PINK1 (ii) and DJ-1 (iii) levels in the control (ctrl) sgRNA and indicated knockout (KO) of NTera2 DNLCs immunoblotted with anti-NENF, PINK1, or DJ-1 antibodies. Band intensities normalized to GAPDH loading control (LC).
